# Conquering oncogenic KRAS and its bypass mechanisms

**DOI:** 10.7150/thno.71260

**Published:** 2022-07-18

**Authors:** Pingping Hou, Y. Alan Wang

**Affiliations:** 1 Center for Cell Signaling, Rutgers New Jersey Medical School, Newark, New Jersey 07103, USA; 2 Department of Microbiology, Biochemistry and Molecular Genetics, Rutgers New Jersey Medical School, Newark, New Jersey 07103, USA; 3 Rutgers Cancer Institute of New Jersey, New Brunswick, NJ 08903, USA; 4 Department of Cancer Biology, The University of Texas MD Anderson Cancer Center, Houston, Texas 77030, USA; 5 Lead contact

**Keywords:** KRAS, RAF, MEK, targeted therapy resistance, cancer

## Abstract

Aberrant activation of KRAS signaling is common in cancer, which has catalyzed heroic drug development efforts to target KRAS directly or its downstream signaling effectors. Recent works have yielded novel small molecule drugs with promising preclinical and clinical activities. Yet, no matter how a cancer is addicted to a specific target - cancer's genetic and biological plasticity fashions a variety of resistance mechanisms as a fait accompli, limiting clinical benefit of targeted interventions. Knowledge of these mechanisms may inform combination strategies to attack both oncogenic KRAS and subsequent bypass mechanisms.

## Introduction

The oncogenic function of *RAS* was first discovered in 1982, when three groups independently found that genomic DNA from cancerous cells contains homologues of the viral *ras* genes (see review 1). Later,* HRAS* and *KRAS* were discovered in several carcinogen or radiation-induced mouse cancer models. In 1984, *KRAS* G12R mutation was identified first in human lung cancer. Soon after, frequent KRAS G12 mutations were confirmed in various human cancers including pancreatic ductal adenocarcinoma (PDAC), colorectal cancer (CRC) and non-small cell lung cancer (NSCLC). The genetically engineered mouse (GEM) models generated since late 1980s further support the oncogenic role of *RAS* in tumor initiation and maintenance.

RAS proteins are small GTPases that are in either active GTP-bound state or inactive GDP-bound state regulated by large multi-domain protein complexes - guanine nucleotide exchange-factors (GEFs) including SOS family members and GTPase-activating proteins (GAPs) such as NF1 2. While GAPs facilitate RAS GTPase to hydrolyze GTP into GDP leading to RAS inactivation, GEFs dislodge bound GDP for GTP to activate RAS. Importantly, the regulation of RAS by GEFs and GAPs depends not only on upstream signal inputs such as receptor tyrosine kinases (RTKs), G-protein coupled receptors (GPCRs) and integrins, but also by their proximal localization on cell membrane. Aberrant RAS hyperactivation can be achieved by gain-of-function mutations of RTKs that assemble several scaffolding proteins to stimulate RasGEFs, loss-of-function mutations of RasGAPs, and RAS oncogenic mutations. In contrast to wildtype protein, oncogenic RAS mutants have impaired GTPase activity, such as the two mutation hot spots glycine 12 and 13 (G12/13) and glutamine 61 (Q61), that keeps RAS in a constitutively active state 3-6. Other factors that regulate RAS signaling transduction include posttranslational modifications and RAS dimerization 7. Though dissociation of mutant KRAS homodimer results in tumor suppression, blocking heterodimerization between wildtype and mutant KRAS accelerates cancer cell growth in lung cancer models 8.

Oncogenic KRAS regulates almost all the cancer hallmarks. KRAS promotes Cyclin D1-dependent cell proliferation, suppresses apoptosis by promoting the expression of BCL-2 family proteins, rewires cancer metabolism covering glycolysis, glutaminolysis, macropinocytosis, mitophagy, redox balance, and macromolecule biosynthesis of amino acids, nucleotides and fatty acids (see review 9), remodels tumor microenvironment (TME) to facilitate tumor growth 10-13, and protects tumor cells from immune surveillance 10, 14-17.

## The KRAS-RAF-MEK cascade

KRAS mediates mitogenic signal transduction from cell surface receptors to intracellular effectors and pathways, including RAF-MAPK, PI3K-AKT, RalGEF-Ral-TBK1, TIAM1-Rac, PLCε-PKC, AF6, RIN1 and RASSF proteins (see review 18-20). Upon binding of ligands to these RTKs (e.g., EGFR, FGFR, PDGFR, IGFR, etc.), RasGEFs activate KRAS, which recruits Raf proteins (A-raf, B-raf and C-Raf) to cell membrane, and thus leading to Raf activation 21, 22. C-raf is the first identified *bona fide* KRAS effector. As serine/threonine protein kinases, Raf proteins phosphorylate mitogen/extracellular protein kinases (MEK1 and MEK2), subsequently leading to the phosphorylation of the extracellular signal-regulated kinases (ERK1 and ERK2) by MEK at threonine and tyrosine residues. Phosphorylated ERK1 and ERK2 translocate to nucleus to activate transcription factors such as ternary complex factors (TCFs), MYC and AP-1 that promote cell cycle progression. In addition, ERK has over 200 targets contributing to cell survival, growth, metabolism and mobility (see review 23). Due to the central role in regulating fundamental cellular processes, the RTK-RAS-MAPK pathway is the most frequently altered signaling pathway in 46% of all cancer types in the Cancer Genome Atlas (TCGA) datasets, and KRAS is mutated in 9% cancer cases with high frequency in PDAC (72%), genomically stable CRC (69%), and NSCLC (33%) 24.

The regulation of KRAS-RAF-MEK signaling pathway has been extensively studied in cancer and significant progress has been made in targeting the cascade. This review will introduce current progress in inhibitors and novel therapeutic approaches targeting KRAS, RAF and MEK, summarize resistance mechanisms and discuss strategies to overcome resistance.

### Targeting KRAS-RAF-MEK signaling

#### KRAS inhibition

Efforts in chemically targeting KRAS were largely unsuccessful in the past 30 years due to, but not limited to, its picomolar affinity for GTP/GDP that restricts the usage of GTP analogues to compete binding with the catalytic domain 25, 26, the lack of known allosteric regulatory sites that increases the difficultness in exploring drug docking pockets 27, and the alternative post-translational modifications of KRAS to ensure correct membrane localization such as geranylgeranylation that escapes the blockade of farnesyl transferase 28. Inhibition of wild type KRAS also raises concerns about potential adverse events (AEs) regarding the importance of KRAS signaling in normal cells. Thus, targeting specific KRAS mutants would be ideal, and, in fact, great progress has been made in recent years (**Table 1**).

*KRAS small molecule inhibitors.* A breakthrough came in 2013, when the Shokat group exploited the KRAS G12C mutation by a disulphide-fragment-based “tethering” screening against KRAS-GDP and discovered the first covalent inhibitor specifically relied on KRAS G12C mutation and GDP-bound inactive state 29. A new pocket under the effector binding switch-II region of KRAS G12C facilitates the inhibitor binding that causes the GDP-favorable structure remodeling. Inspired by the pioneering work, high affinity KRAS G12C inhibitors (G12Ci) are developed by several groups 30-34, which share a common trapping mechanism that locks KRAS G12C in a constitutively inactive state 35. The first-in-class G12Ci with clinical activity are listed in **Table 1**, among which sotorasib (a.k.a LUMAKRAS or AMG 510) received FDA approval recently.

The phase 1 clinical trial data of sotorasib shows 32.2%-37.6% objective response rate (ORR) and improved median progression-free survival (PFS) of 6.3-6.8 months as compared with 2.5-4 months under standard of care (SOC) in heavily pretreated NSCLC patients, while CRC patients only exhibits 7.1% ORR and median PFS of 4 months as compared with 1.9-2.1 months under SOC 36, 37. Similarly, NSCLC patients respond to G12Ci adagrasib (MRTX849) better than CRC patients (43% versus 25% ORR). Thus, understanding the different tumor responses to G12Ci in individuals with the same cancer type or with distinct cancers are needed to expand clinical applications and enhance the anti-tumor effect of G12Ci. In addition, to increase the efficacy, clinical trials evaluating combinations of G12Ci with chemotherapy reagents, immune checkpoint blockade such as PD-L1/PD-1 antibodies, and inhibitors of SHP2, tyrosine kinases and MEK are ongoing. These treatment regimens are designed to either enhance the KRAS vertical pathway inhibition or leverage the increased T cell infiltration upon G12Ci treatment, which are discussed in the next section. Other KRAS inhibitors (KRASi) under pre-clinical evaluation include G12Ci BI 1823911, G12D inhibitors MRTX1133 and BI-KRASG12D1, RAS G12C(ON) inhibitor RMC-629 and pan-RAS (ON) inhibitor RMC-6236. In contrast to current G12Ci, RMC-629 and RMC-6236 interact with and inhibit active RAS by forming a RAS-inhibitor-chaperone protein tri-complex, which may overcome resistance induced by active residue RAS.

*Exosome-delivered KRAS siRNA*. Compared to artificial carriers such as liposomes, natural carrier exosomes express CD47 immunoglobulin that avoids phagocytosis by macrophages to prolong the half-life of delivered drugs 38. Recent pre-clinical studies revealed that fibroblasts-derived exosomes loaded with G12D siRNA (iExosomes) efficiently attenuate PDAC tumor growth and extend tumor bearing mouse survival 38, 39. The efficacy of the iExosomes is currently being evaluated in a phase 1 clinical trial. However, AE and “off-target” concerns remain to be addressed since exosome-delivered reporter was also observed in liver and spleen 39.

*mRNA vaccines*. Though the first *in vivo* test was reported in 1990, mRNA vaccine has become a feasible and promising therapeutic strategy in recent years as the instability, high immunogenicity and inefficient mRNA delivery *in vivo* have been improved 40. The KRAS mRNA vaccine V941 (mRNA-5671) co-developed by Moderna and Merck is in phase 1 clinical trials. Lipid nanoparticle encapsulated mRNA vaccines encoding mutant KRAS neoepitopes (G12D, G12V, G13D and G12C) are taken up and translated in antigen presenting cells, and then presented by MHC molecules on cell surface. However, KRAS mutant cancer cells may still evade neoantigen-specific T cell immune surveillance by suppressing antigen presentation machinery 41-43 and inflammatory response in TME 16, 43.

*Anti-KRAS T cell transfer.* Adaptive cell therapies, such as tumor-infiltrated lymphocytes (TILs) therapy, engineered T cell receptor (TCR) therapy and chimeric antigen receptor T cell therapy, have made impressive progress in melanoma, sarcoma and B cell lymphomas in the last decade 44-47. HLA-A*11:01-restricted human KRAS G12D or G12V-reactive TCRs are identified in immunized HLA-A*11:01 transgenic mice. Peripheral blood lymphocytes (PBL) transduced with these TCRs can recognize several HLA-A*11:01-positive KRAS mutant human PDAC cell lines and suppress xenograft tumor growth 48. Based on these results, phase 1 clinical trials utilizing autologous PBLs transduced with HLA-A*11:01-restricted murine TCR (mTCR) recognizing human KRAS G12D or G12V are ongoing now. A promising clinical result from a TILs transfer therapy in 2016 showed that infusion of TILs specifically against KRAS G12D in a metastatic CRC patient resulted in tumor regression in all seven metastatic lung lesions for 40 days, followed by one lesion progression after 9 months 49. Four distinct TCRs were identified that recognize two KRAS G12D neoantigens, a 9mer and a 10mer, which are all restricted by human leukocyte antigen HLA-C*08:02 49, 50. Importantly, both KRAS G12D and HLA-C*08:02 are required to generate the recognizable neoantigens 50. Though not as common as HLA-A*11:01, 2.5-10% KRAS G12D patients have HLA-C*08:02 allele 51, who would potentially benefit from the TCR therapy.

*KRAS antibody.* While traditional antibodies target extracellular proteins, a new type of antibodies is internalized via endocytosis and subsequent endosomal escape to target intracellular proteins 52, 53. The second generation of cell permeable RAS antibody in human IgG1 format (pan-RAS iMab, inRas37) showing favorable PK *in vivo* with half-life of 3.5 days, only interacts with ATP-bound active RAS mutants but not wild type RAS, attenuates ERK and AKT signaling and suppresses tumor growth in several KRAS mutant (G12D, G12V, G13D, etc.) xenograft models 52. Whether the KRAS antibody will be effective in cancer patients needs to be explored.

*Targeting RBD domain of RAS effectors.* RAS effector proteins interact with RAS through RAS-binding domain (RBD). Rigosertib is a RAS mimetic that interacts with RBDs of RAF, PI3K and Ral-GDS to interrupt their RAS binding 54. Rigosertib could effectively block RAS-driven fibroblast cell transformation and suppress xenograft tumor growth 54. The efficacy of rigosertib and nivolumab is evaluated in a phase 1 clinical trial in KRAS mutant NSCLC patients with advanced disease as a second-line treatment.

#### RAF inhibitors

Though wildtype RAF inhibitors show limited anti-tumor activity in KRAS or BRAF mutant cancers in clinical trials, ATP-competitive RAF inhibitors that selectively target BRAF V600E monomers such as vemurafenib, dabrafenib and encorafenib increase clinical benefit, but they often paradoxically activate ERK signaling by transactivation of the other protomer in RAF dimers, which eventually results in drug resistance. Combination therapy of BRAF V600E inhibitor with MEK or EGFR inhibitors enhances the efficacy compared to monotherapy and prolongs the overall survival of CRC and melanoma patients 55-58. In addition, the next-generation RAF inhibitors can block BRAF monomers and dimers via two distinct mechanisms. BRAF inhibitors such as LY3009120, BGB-283 and LXH254 equipotently inhibit both protomers in RAF dimers without paradoxical activation of ERK 59, 60. Alternatively, RAF inhibitors including PLX7904 and PLX8394 can disrupt BRAF homo- or hetero-dimers as “paradox breakers”, but not CRAF dimers 61. Though these inhibitors show great efficacy in preclinical models, clinical efficacy varies. BGB-283 and LXH254 monotherapy resulted in objective responses in BRAF mutant patients in phase 1 clinical trials 62, 63, while LY3009120 or PLX8394 monotherapy lacked responses 64, 65. Combined PLX8394 and cobicistat treatment is promising that 42% and 65% PR are observed in BRAF V600E CRC and glioma patients, respectively 65. Taken together, whether the next-generation BRAF inhibitors perform better and exhibit broader anti-tumor responses than the first-generation inhibitors need further clinical evaluation.

##### MEK inhibitors

MEK inhibitors trametinib, cobimetinib, binimetinib and selumetinib are the four FDA-approved drugs to date. Additionally, there are more than a dozen MEK inhibitors in clinical trials and about 8 inhibitors under preclinical development 66. Most of the MEK inhibitors are with high specificity and strong binding affinity to MEK kinase because they dock in an allosteric pocket near catalytic site that causes conformational changes and subsequent blockage of kinase activity. The fact that MEK inhibitors only have modest clinical efficacy in RAS mutant cancers compared to BRAF mutant cancers is mainly due to the relief of ERK-mediated negative feedback and the BRAF, CRAF-dependent reactivation of MEK 67, 68. Thus, combined inhibition of MEK and their upstream kinases (e.g., RAF, RAS, EGFR, etc.) are expected to enhance MAPK signaling inhibition and show better clinical efficacy than monotherapy in RAS mutant cancers, as did in clinical trials now.

### Resistance mechanisms to KRAS-RAF-MEK signaling inhibition

**Figure 1:**
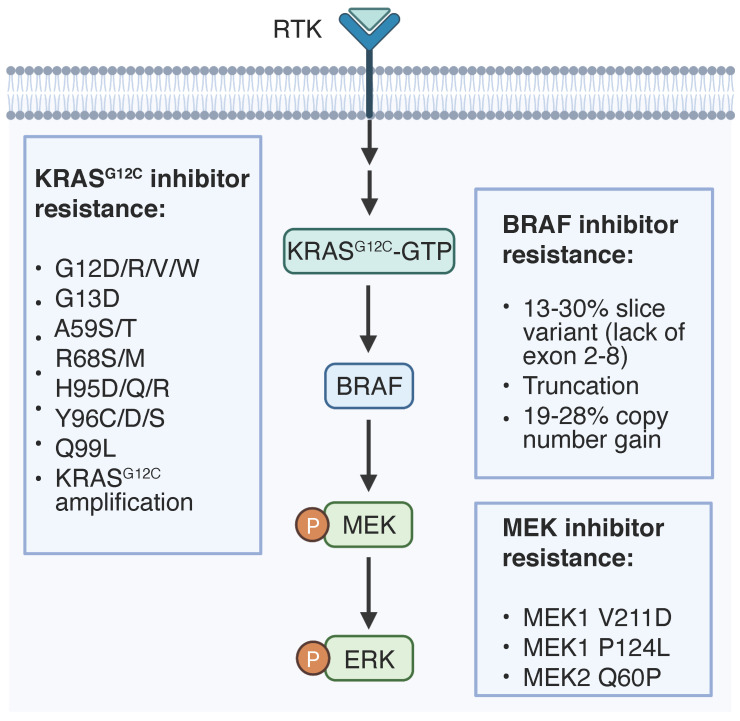
** On-target resistance mechanisms to RAS signaling inhibition.** Genetic alternations of targeted proteins that regulate therapy resistance are listed.

**Figure 2:**
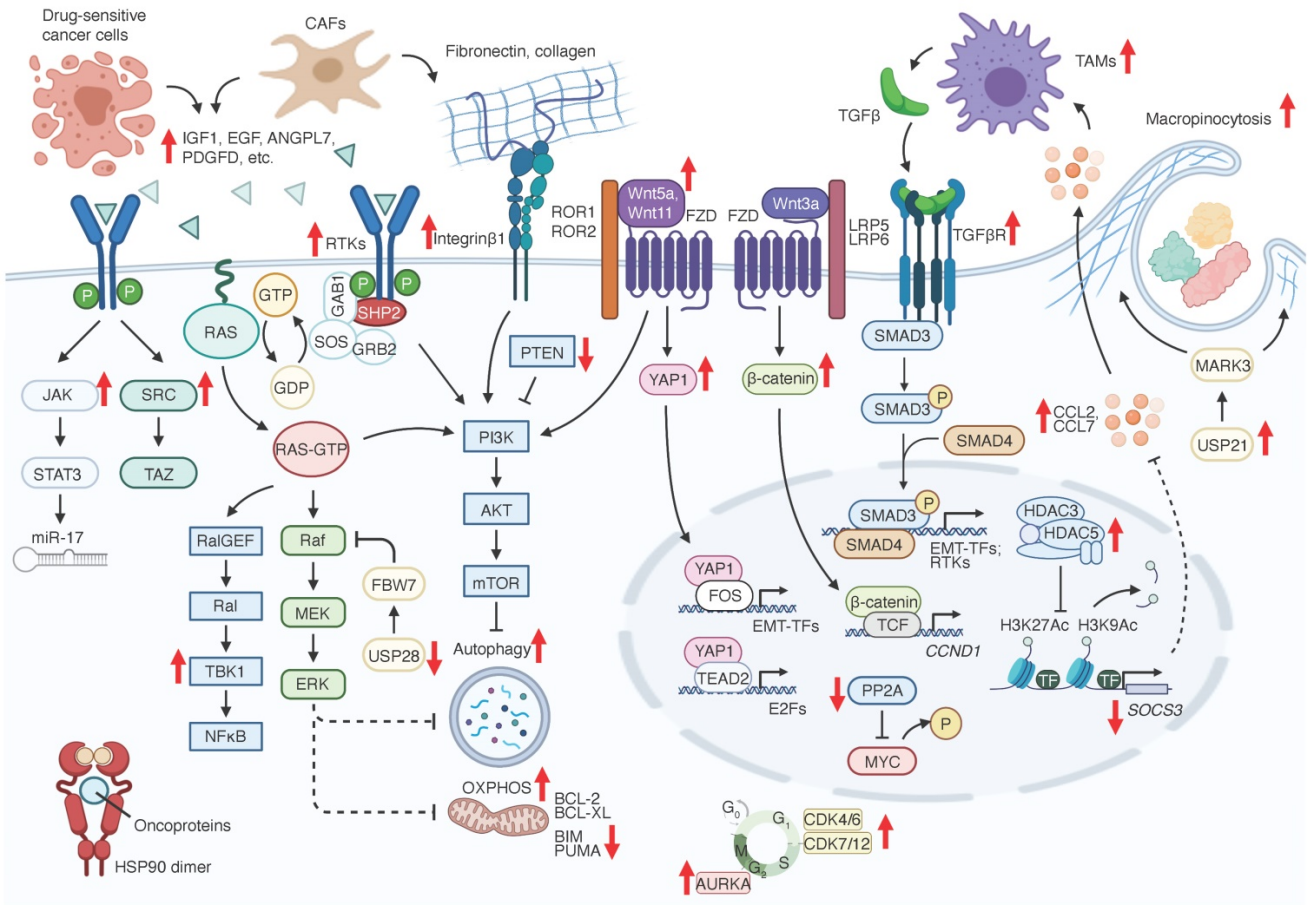
** Adaptive resistance mechanisms to RAS signaling inhibition.** Bold red arrowheads indicate major factors that either promote (up) or suppress (down) KRAS signaling inhibition resistance.

Given the recent development of agents targeting Kras pathways and their clinical application, it is paramount to understand the various therapeutic resistance mechanisms and develop better agents to overcome them. Resistance mechanisms to KRAS-RAF-MEK signaling inhibition include genetic alterations of targeted oncogenes (**Figure 1**) and adaptive activation of alternative regulators or signaling pathways in tumor cell intrinsic and extrinsic manners (**Figure 2** and **Table 2**).

#### On-target resistance

#### Second-site mutations

Site-mutations could confer resistance to G12Ci 69-72 and allosteric MEK inhibitors (MEKi) 73, 74. Resistance mutations in KRAS (G12C/V, G13D, Y96D and Q61L/R/K) have been detected in cell-free DNA of a NSCLC patient post G12Ci adagrasib treatment 70. A larger study evaluating adagrasib in 38 patients also identified several KRAS mutations (G12D/R/V/W, G13D, Q61H, R68S, H95D/Q/R, Y96C) associated with drug resistance 71. Similarly, a mutation screening in lung cancer cells revealed second-site KRAS mutations that confer resistance to sotorasib and adagrasib (Y96D/S), sotorasib only (G13D, R68M, A59S and A59T) and adagrasib only (Q99L) 69. Mechanistically, switch II pocket mutations (R68S, H95D/Q/R and Y96C/D/S) interferes the binding of G12Ci to inactive KRAS, while a RAS G12C(ON) inhibitor or dual inhibition of SOS1 and MEK1 may overcome the G12Ci resistance 69, 70.

Resistance-related mutations of MEK1 favor hydrophobic drug docking pocket to directly interrupt drug interaction (e.g., V211D), or the N-terminal negative regulatory helix (helix A) to upregulate intrinsic MEK1 kinase activity (e.g. P124L). MEK1 P124L mutation was observed in a metastatic tumor biopsy from a drug resistant melanoma patient upon MEKi AZD6244 treatment, while dual inhibition of MEK and BRAF can overcome the acquired resistance 73. MEK1 V221D mutation, which is localized within the arylamine binding pocket, emerged in a CRC patient following the treatment with MEKi binimetinib and anti-EGFR antibody panitumumab 74. MEK1 V211D reduces drug affinity of all the available allosteric MEKi, leading to MEK1 hyperactivation, but it is sensitive to an ATP-competitive MEKi MAP855 74. MEK2 Q60P mutation was identified as a resistance driver in 3 out of 5 BRAF mutant melanoma patients under dual inhibition of MEK and BRAF, while ERK inhibitors can attenuate the elevated ERK signaling and reverse the refractory phenotype 75. Collectively, second site mutations are predominant resistance mechanisms that require follow up targeted genomic sequencing for cancer patients under such regimes.

#### Splice variants

An internal splice variant of BRAF V600E, which lacks exons 2-8, a region encoding RBD, is recurrently observed in treatment refractory melanoma patients to RAF inhibitor (RAFi) at a frequency of 13-30% 76-80. In comparison to full length protein, the truncated BRAF V600E tends to form homodimer and activates MAPK signaling regardless of RAFi 76, 81, blocking of which can abolish persistent ERK activation and sensitize tumor cells to RAFi. Alternatively, S729 phosphorylation in truncated BRAF V600E enhances the interaction with MEK1/2 to confer RAFi resistance 79, thus MEKi combination may overcome it. In-frame insertion of three residues on ɑC-β4 loop of BRAF in cancers assembles a large hydrophobic network that involves in R-spine, which impairs BRAF dimerization and MEK inhibitor association 82. Thus, these splice variants are sensitive to MEK inhibitors albeit resistant to BRAF inhibitors. In-frame deletions of the β3-ɑC loop of MEK1 can also enhance MEK1 homodimerization, enforce ERK activation and drive resistance to MEK inhibitors 81.

#### Amplification

High-level amplification of KRAS G12C allele has been observed in 2 out of 17 ctDNA samples from cancer patients who are refractory to adagrasib 71. Whole exome sequencing of drug resistant tumors revealed the recurrent copy number gain of BRAF V600E at a frequency of 19%-28% as a common resistant mechanism to RAFi in melanoma, which hyperactivates ERK signaling independent of CRAF 80, 83-85. Similarly, acquired focal BRAF V600E amplification was found in a BRAF mutant CRC patient whose disease progressed to triple inhibition of RAF, EGFR and MEK 86. Importantly, these non-responder tumor cells are still addicted to KRAS-RAF-MAPK cascade and sensitive to inhibitors targeting the downstream factor ERK.

#### Off-target resistance

##### Genetic alterations in vertical RTK-RAS-MAPK pathway

*KRAS inhibition resistance.* Recent clinical data reveal acquired resistance mechanisms of adagrasib included MET amplification, activating mutations in NRAS (Q61K and G13R), MRAS (Q71R), BRAF (V600E and G596R) and MAP2K1 (K57T/N), oncogenic fusions involving ALK, RET, BRAF, RAF1, and FGFR3, and loss-of-function mutations in NF1 and PTEN 71, 72, all converging on RAS signaling pathway reactivation.

*RAF inhibition resistance.* Off-target MAPK vertical pathway alterations account for 52% BRAFi resistance in 71 melanoma patients, including NRAS mutations (G12D/R, G13R, and Q61K/R/L) at 18%, KRAS mutations (G12C, G12R, and Q61H) at 7% and MAP2K1 mutations at 3% (K57N and C121S) 80. In addition, amplifications at EGFR, BRAF V600E, NRAS and KRAS loci, mutations of NRAS, KRAS and MAP2K1, and PTEN loss are often observed in resistant lesions of melanoma and CRC patients with treatment of combined RAF targeted therapies 85-87. NRAS can enhance BRAF and CRAF interaction to activate ERK signaling in RAFi-refractory cells 83, 88. Therefore, inhibitors targeting RAF dimers, CRAF or MEK may overcome the resistance.

*MEK inhibition resistance.* Three independent *in vitro* studies all observed BRAF or KRAS amplification in MEKi resistant CRC and breast cancer cells 89-91. Since these cells still addict to MAPK signaling, dual inhibition of MEK and BRAF (or ERK) is effective to reverse resistance.

##### Epigenetic modulators

*Writers.* HAT1 acetylates newly synthesized free histone H4 in cytoplasm, whose function is controversial in different cancer models 92-95. HAT1 downregulation is associated with non-responsiveness of BRAFi and MEKi in melanoma patients, and HAT1 depletion drives resistance to BRAFi vemurafenib and dabrafenib by upregulating MAPK via IGF1R in melanoma cells 96. Histone methyltransferase SMYD3 methylates MAP3K2 at lysine 260 that blocks PP2A interaction in KRAS mutant PDAC and NSCLC cells, followed by activation of MAPK signaling 97. Thus, knockout of SMYD3 could potentiate MEKi to impair KRAS-driven pancreatic neoplasia. However, whether HAT1 loss confers BRAFi resistance *in vivo* and whether SMYD3 is a potential therapeutic target to prevent KRASi resistance still need further investigation.

*Erasers.* Histone deacetylase (HDAC) family members have been extensively studied in therapy resistance 98-101. Depending on sequence homology and domain structures, HDACs are classified into four classes: I (HDAC1, 2, 3, 8), IIA (HDAC4, 5, 7, 9), IIB (HDAC6, 10), III (sirtuins) and IV (HDAC11). Pan-HDAC inhibitors potentiate the cytotoxic effect of MEKi in human PDAC, uveal melanoma and CRC cells 98-100, and one possible mechanism is that HDAC inhibitors attenuate treatment-induced upregulation of pAKT and YAP1 100. Functional studies in PDAC cells reveal that HDAC4 and HDAC6, instead of HDAC1, likely rescue the pro-apoptotic phenotype induced by dual inhibition of HDAC and MEK 99. However, HDAC class I, HDAC6 or HDAC8 specific inhibitors are less effective for tumor suppression compared to pan-HDAC inhibitors 100, suggesting the functional redundancy among HDACs.

SETD5, a scaffold of HDAC3-G9a co-repressor complex, drives trametinib resistance in PDAC cells by upregulating genes in cytochrome P450 and glutathione metabolism pathways 101. HDAC5, a scaffold of HDAC3 corepressor complex, is the only HDAC promoting PDAC cells to bypass KRAS dependency 102. HDAC5-HDAC3 corepressor complex suppresses *Socs3* expression via deacetylation of H3K9 and H3K27, resulting in chemokine reprogramming and followed by TME remodeling. TGFβ provided by tumor associated macrophages (TAMs) is the key driver of the KRAS bypass. In summary, HDACs may play distinct or redundant roles in regulating therapy resistance in a context-dependent manner. Considering that HDAC inhibitors may also affect cells of the TME such as reprogramming TAMs 103, comparing the efficacy of HDAC pan- and specific inhibitors in cancer mouse models *in vivo* is essential.

*Readers.* Bromo and Extra Terminal Domain (BET) family proteins include BRD2, BRD3, BRD4 and BRDT that recognize acetylated lysine residues for protein-histone association and chromatin remodeling 104. The tumoricidal activity of BET inhibitors (BETi) varies in solid tumors, while genetic analysis has identified that KRAS G12 mutations are significantly associated with BETi resistance 105. Synergy of MEKi and BETi is observed in RAS hyperactivated CRC, triple-negative breast cancer (TNBC), multiple myeloma (MM) and PDAC 105, 106, and is explained by suppression of adaptive epigenetic reprogramming 107. Compared to monotherapy, combinatory treatment widely downregulates mitotic genes, upregulates apoptotic genes, sustains pERK inhibition and causes cell cycle arrest 105. In TNBC models, BETi JQ1 disrupts enhancer remodeling induced by MEKi trametinib, blocks kinome reprogramming and suppresses tumor growth; in lung cancer models, JQ1 can overcome MEK and TBK1 inhibition resistance via suppressing adaptive activation of YAP1/TAZ and IGF1/IGF1R signaling 108.

Tumor genetic heterogeneity regulates tumor vulnerabilities to dual inhibition of MEK and BET. Homozygous loss of PRC2 components SUZ12 and EED is frequently observed in malignant peripheral nerve sheath tumors (MPNSTs) with NF1 microdeletion 1. SUZ12 loss amplifies Ras-driven transcription by promoting an epigenetic switch from H3K27me3 to H3K27Ac. JQ1 suppresses PRC2-regulated expression of RAS signature genes and synergizes with MEKi to severely impair MPNST cell growth. In addition, PRC2 dysregulation increases HOXC10 expression in KRAS mutant NSCLC, which coordinates with MEK to enhance the expression of E2F1 targets encoding pre-replication complex proteins 109. Simultaneous deletion of NF1 and SUZ12 is presented in 24% melanoma and 14% GBM cases and dual inhibition of MEK and BET induces the genetic context-specific replication fork stalling and DNA damage, highlighting the potential therapeutic benefit of the combinatory treatment.

##### Receptor tyrosine kinases

RAS or RAF oncogenic alterations hyperactivate MAPK signaling regardless of upstream inputs, while pharmacological inhibition of RAS-RAF-MAPK suppresses the negative feedback on RTKs. The preferential upregulation of specific RTKs and their ligands responded to RAS signaling inhibition varies 110-112, resulting in cancer type or subtype-specific combinatory strategies to overcome RAS signaling inhibition resistance.

*ErbB receptor family.* The ErbB receptor family is composed of four members: ERBB1/EGFR, ERBB2/HER2, ERBB3/HER3 and ERBB4/HER4, which mainly activate MAPK and PI3K-AKT signaling pathways. High EGFR promotes RAFi resistance in CRC, lung, melanoma and thyroid cancer cells by rapid feedback activation of EGFR signaling 113-117, and determines the G12Ci refractory state in KRAS G12C cancers 33, 34, 118-120. However, EGFR does not seem to be the dominant driver of MEKi resistance in PDAC. Blocking all activated RTKs, including AXL, PDGFRα, EGFR and HER2, is required to further impair tumor growth with MEKi 121, suggesting functional redundancy of these RTKs in PDAC. In addition, increased autocrine secretion of HER3 ligand Neuregulin-1 and the upregulation of HER3 lead to MAPK reactivation upon RAFi in thyroid cancer cell lines 122. Targeting HER3-EGFR and HER3-HER2 heterodimers can reverse MEKi non-responsiveness in KRAS mutant NSCLC and CRC cells where MYC activates HER3 123.

*PDGF receptor family.* PDGF receptors are engaged by homo- or heterodimers of PDGF-A, -B, -C and -D. PDGFRA and PDGFRB are homo- or heterodimerized depending on bound PDGFs to activate downstream signaling. Upregulation of PDGFRB drives RAFi resistance in a subset of melanoma cell lines which have low RAS and MAPK activities 124. Another study in PDAC revealed PDGFRA as a driver of MEKi resistance via JAK-STAT3 activation 125.

*FGF receptor family.* The FGF receptor family comprises FGFR1, 2, 3 and 4 that interact with their ligands to activate signaling pathways including RAS-MAPK. FGFR mediates refractory to G12Ci in PDAC and NSCLC 118. Depletion of FGFR1 sensitized tumor cells to trametinib in KRAS mutant NSCLC and PDAC, but not CRC 126. MEKi activates FGFR1 signaling via downregulation of SPRY4 to protect tumor cells from death by elevating AKT survival signal 127.

*IGF1 receptor.* IGF1R regulates resistance to chemotherapy 128, radiotherapy 129 and EGFR targeted therapy 130. BRAFi resistant melanoma cells upregulate several RTKs, but only co-targeting IGF1R and MEK exacerbates cell death 131. Moreover, dual inhibition of IGF1R and KRAS signaling (MEK, mTOR and G12C) markedly enhances anti-tumor effects in CRC and NSCLC cells 132-134. IGF1R also promotes PI3K activation and KRAS depletion resistance in a group of KRAS mutant CRC cell lines 135.

*MET receptor.* MET is a proto-oncogene encoding c-Met that binds hepatocyte growth factor (HGF) to activate signaling pathways, including MAPK, PI3K, SRC and STAT 136. MET amplification is a major resistance mechanism to EGFR inhibition in lung cancer 137. MET depletion suppresses ERK phosphorylation and overcomes BRAFi resistance in melanoma cells 138. Moreover, *MET* is recurrently amplified in relapsed tumors after genetic depletion of BRAF in anaplastic thyroid carcinoma models, which elevates MAPK signaling and is essential for resistant cell growth 139.

*SHP2/PTPN11.* SHP2 is an allosteric protein-tyrosine phosphatase (PTP) that is released from autoinhibition by tyrosine phosphorylation upon cytokine or growth factor stimulation 140. SHP2 inhibition (SHP2i) blunts GAB1-GRB2-SOS1 complex formation assembled by RTKs, thus interfering with RAS-GTP loading and activation 141. Increased RAS-GDP occupancy provides a unique vulnerability to KRAS-GDP bound G12Ci, and complete engagement of KRAS G12C by ARS-1620 was observed in 1 hour compared to about 70% basal engagement rate in cells 142. SHP2i enhances the efficacy of G12Ci, exacerbates MAPK suppression and overcomes G12Ci resistance in various cancers 33, 112, 118, 143-146. Though RAS/RAF-dependent cancer cells are refractory to SHP2i monotherapy 147, SHP2i blocks EGFR-mediated MAPK rebound and confers sensitivity in BRAFi resistant CRC cells 138, 148, and synergizes with MEKi to suppress PDAC and NSCLC growth 149, 150.

*SHOC2.* The SHOC2-PP1c holoenzyme is composed of SHOC2 scaffold protein and the catalytic subunit of protein phosphatase 1 (PP1c), an effector of MRAS essential for growth factor-mediated RAS signaling activation 151. Depletion of SHOC2 blocks RTK-mediated ERK activation and causes synthetic lethal with MEKi in KRAS mutant PDAC and NSCLC cells 111 and with RAFi in BRAF mutant CRC cells 138.

##### Other kinases

*TBK1.* TBK1 is an atypical IκB kinase family member that is directly recruited and activated by the RalB/Sec5 effector complex 152. RalB-TBK1 activation is regulated by RalGEFs with RAS association domain or RAS-independent RalGPS1 153. TBK1 promotes cancer cell proliferation via CCL5 and IL-6 mediated STAT3 activation, and inhibition of TBK1 overcomes MEKi resistance in KRAS mutant NSCLC and NRAS mutant melanoma cells 154, 155.

*PP2A.* PP2A, a serine/threonine phosphatase in the form of trimeric protein complex, is a putative tumor suppressor, inhibition of which facilitates RAS-induced human cell transformation 156. Depletion of PP2A results in MEK/ERKi resistance in KRAS mutant NSCLC by upregulation of AKT-mTOR signaling and MYC, while PP2A activator DT-061 and MEKi synergistically impair NSCLC growth 157.

*CDKs.* Targeting KRAS, BRAF or MEK alone mainly exerts cytostatic rather than cytotoxic effects in cancer cells 145, 158-162. CDK7/12 inhibition attenuates MEKi induced transcriptional and enhancer remodeling, resulting in enhanced cytotoxicity and tumor regression in NSCLC, melanoma, and gastric cancer models 163. In addition, CDK4/6 inhibitors exhibit synergistic cytotoxicity with G12Ci across multiple cancer types 33, 34, 118, 143.

*SRC family kinase.* Src family kinases, which are often overexpressed in cancers, regulate various biological events including gene transcription, cell adhesion, invasion, proliferation, survival and angiogenesis 164. Src inhibitor dasatinib synergizes with trametinib to induce cell cycle arrest and apoptosis by downregulating TAZ in KRAS-mutant cancer cells, but not in wild type KRAS or EGFR mutant cancer cells 165.

*COT/TPL2.* COT kinase is encoded by MAP3K8 that activates MAPK signaling independent of RAF. Enforced expression or amplification of *MAP3K8* promotes BRAFi and MEKi resistance in melanoma cells, and MAP3K8 upregulation is observed in a group of disease-progressed melanoma patients on BRAFi or MEKi treatment 166. As required for resistant melanoma cell survival, MAP3K8 is a potential therapeutic target in this specific setting.

*Aurora A kinase.* Aurora A belongs to a family of mitotic serine/threonine-protein kinases that regulates cell division 167. AURKA is upregulated in G12Ci resistant cells, depletion of which augments the antiproliferative effect of ARS-1620 158. Aurora A interacts with KRAS G12C-CRAF complex in lung cancer cells, and dual inhibition of G12C and aurora A dissociates the complex resulting in a long-term MAPK signaling inhibition and the prevention of tumor relapse.

##### Signaling pathways

*PI3K/AKT/mTOR.* MEKi reactivates PI3K in PDAC models 121, 161. Though ineffective alone, targeting AKT synergizes with MEKi to suppress PDAC growth 121, 161, 168. Conversely, forced PI3K and AKT hyperactivation promotes KRAS bypass in PDAC 121, 169. Clinical data analysis reveals that ~4% BRAFi unresponsive melanoma patients gain nonsynonymous mutations of PTEN, PI3K and/or AKT 80. In CRC, elevated AKT signaling is associated with tumor intrinsic MEKi resistance 170, 171. In addition, PI3K inhibitor could shift IC50 of ARS-1620 to over 2-fold lower compared to monotherapy, overcome G12Ci resistance and retard tumor growth in NSCLC 110, 143. Consistently, mTOR inhibitor enhances tumoricidal effect of adagrasib 33.

*JAK-STAT.* STATs activate genes related to cell proliferation, differentiation, and apoptosis, providing the basis for regulating tumor response towards drug treatments. A pilot study of 38 NSCLC lines identified that JAK-STAT activation is correlated with resistance to AZD6244 172. STAT3 inhibition enhances AZD6244-induced cell apoptosis by downregulation of miR-17 and induction of BIM, while activation of STAT3 elicits AZD6244 resistance. The autocrine activation of STAT3 upon MEKi is mediated by FGFR-PI3K and JAK kinases in KRAS mutant NSCLC but not in CRC or KRAS wild-type NSCLC 173, suggesting the context-dependent engagement of STAT3 feedback loop.

*Wnt.* Both canonical and non-canonical Wnt pathways regulate cancer stem cells (CSCs), which are often accounted for treatment refractory and tumor recurrence 164. Elevated WNT5A is associated with disease progression and acquired resistance in a subset of melanoma cases under BRAFi 174. Mechanistically, WNT5A binds to its receptors RYK and FZD7 to activate PI3K-AKT signaling. WNT5A also promotes KRAS bypass in PDAC cells by stimulating YAP1 nuclear translocation 175. On the other hand, KRAS mutant CRC cells with high activation of canonical Wnt-β-catenin signaling pathway are refractory to MEKi, inhibition of which overcomes resistance and induces cell apoptosis 176.

##### Other mechanisms

*YAP1.* YAP1 acts as a transcriptional coactivator or corepressor downstream of Hippo pathway that plays an oncogenic role in various cancers 177. YAP1 is negatively correlated with tumor response to BRAFi or RAF/MEK dual inhibition in melanoma patients 178, is recurrently amplified in KRAS-independent relapsed PDAC tumors 179, and is nuclear-localized and activated after KRAS suppression in relapsed lung tumors 180. YAP/TEAD gene signature is also enriched in dormant human NSCLC cells under dual inhibition of EGFR and MEK 181. Functionally, *YAP1* is required for resistance to KRAS signaling inhibition and promotes KRAS independent tumor cell growth 179, 180. Mechanistically, YAP1 cooperates with TEAD2 to activate cell cycle and DNA replication 179, or with the AP-1 transcription factor *FOS* to regulate epithelial-to-mesenchymal transition (EMT) modulators such as *SLUG* 180. Depletion of YAP1 abolishes *SLUG*-mediated repression of pro-apoptotic BMF, leading to enhanced cell apoptosis in combination with MEK and EGFR inhibition 178, 181.

*HSP90.* HSP90, a molecular chaperone, usually plays a critical oncogenic role because many of its clients are signaling transducers and cellular stress responders, and oncogenes hijack its functions to prevent aberrantly expressed or mutant oncoproteins from misfolding or degradation. HSP90 inhibition alone attenuates MAPK signaling and cell growth in RAS or RAF mutant MM cells. Dual inhibition of RAS signaling and HSP90 impairs AKT and ERK activity and amplifies the pro-apoptotic effects 143, 182, 183.

*Deubiquitinases.* Loss of USP28 deubiquitinase stabilizes and upregulates BRAF protein via FBW7, a substrate recognition subunit in the SCF ubiquitin ligase complex, which results in ERK activation and RAFi resistance in melanoma 184. USP21 deubiquitinase not only regulates cancer cell stemness but also drives KRAS bypass in PDAC 185, 186. USP21 can elevate macropinocytosis in KRAS-depleted PDAC cells via deubiquitinating MARK3 to support amino acid homeostasis and activate mTOR signaling pathway.

*Metabolic regulators.* Pyruvate dehydrogenase kinase 4 (PDK4), a gatekeeper of TCA cycle, is downregulated in MEKi resistant NSCLC cells 187, yet the role of metabolic regulators in driving KRAS targeted therapy resistance are still under exploited.

### Salvage pathway activation

#### Protective autophagy

Autophagy is a fine-tuned catabolic program that degrades and recycles damaged proteins and organelles to protect cells from stress induced apoptosis. MEKi, ERKi and KRAS ablation strongly elevate protective autophagy flux via LKB1-AMPK-ULK1 axis, inhibition of which sensitizes tumor cells to KRAS signaling inhibition in PDAC, melanoma and CRC models 162, 188, 189, implying autophagy as a common surviving mechanism.

#### Macropinocytosis

Oncogenic KRAS hijacks macropinocytosis to support the high amino acid demand by PDAC cells 190, 191. KRAS-depleted PDAC cells opt out cell cycle, reduce macropinocytosis and encounter severe metabolic stress. USP21 can partially elevate macropinocytosis by modulating microtubule dynamics, which supports KRAS-independent PDAC cell growth 186.

#### Oxidative Phosphorylation

KRAS drives PDAC tumorigenesis and maintains malignancy partially through regulating glucose uptake and flux into biosynthesis pathways 192. KRAS ablation in PDAC leads to decreased glycolysis and cell death, while a small population of CSCs persist depending upon active oxidative phosphorylation (OXPHOS) 162. OXPHOS inhibitors have been shown to effectivity eliminate the residual surviving cells after KRAS ablation.

#### BCL-2 family proteins

BCL-2 family proteins regulate apoptosis commitment via induction of mitochondrial outer membrane permeabilization and release of pro-apoptogenic factors such as cytochrome c 193. BCL-2 proteins are composed of 3 functionally distinct groups: pro-survival proteins including BCL-2 and BCL-XL, pro-apoptotic pore-formers including BAX and BAK, and pro-apoptotic BH3-only proteins including BIM and PUMA. Adaptive downregulation of BIM and PUMA is observed in MEK and PI3K inhibition resistant NSCLC cells, and triple inhibition of BCL-XL/BCL-2, MEK and PI3K induces cell apoptosis 194.

#### CDKN2A

CDKN2A depletion is recurrently acquired by 7%-28% progressed melanoma patients on treatment with BRAFi or BRAF/MEKi 80, 85, which facilitates tumor cells to overcome cell cycle arrest and subsequent apoptosis.

### Phenotypic dynamics

#### EMT

EMT plays a pivotal role in regulating conventional and targeted therapy resistance in GBM, head and neck squamous cell carcinoma, pancreas, lung, prostate and ovarian cancers 195. The expression of EMT transcription factors (EMT-TFs) and the mesenchymal phenotype are inversely correlated with therapeutic outcome. Induction of EMT by TGFβ overcomes KRAS-MEK dependency in NSCLC through activation of FGFR1 signaling 127, bypasses KRAS dependency in PDAC via activation of MYC and replication-regulatory genes 102 and drives G12Ci resistance by activation of IGFR-IRS1-PI3K pathway in NSCLC 146. EMT-TF *SNAIL*, *SLUG* and *ZEB1* are required for KRAS signaling independence in several NSCLC models 180, 196. Thus, understanding and targeting the EMT program is meaningful for prevention of KRASi resistance.

#### Reversible cytostatic state

Instead of acute cell death, inhibition of KRAS-MAPK signaling in NSCLC, PDAC and melanoma models sequesters a subgroup of tumor cells in a plastic, quiescent, and CSC-like state that enables some of them to gain adaptive changes and become resistance 158, 159, 162, 197. Incomplete KRAS suppression only decreases PDAC cell proliferation in 2-D culture or induces quiescence in 3-D culture while these cells are still able to form tumors *in vivo* 197. Pseudo-time analysis reveals three trajectories representing initial, inhibited, and adapting cell states in G12Ci-treated NSCLC cells, and an elevated quiescent gene signature is observed in most cells 158.

Quiescent G12Ci resistant tumor cells are able to reactivate KRAS signaling and resume proliferation by increasing the synthesis of active KRAS-GTP or stimulating upstream regulators such as EGFR, SHP2 and AURKA 158. OXPHOS is highly relied upon by quiescent PDAC CSCs for energetic metabolism 162. Thus, eliminating quiescent cells by blockage of addicted survival pathways may prolong progression free survival. RAFi induced quiescent melanoma cells express neural crest markers such as NGFR, suggesting a dedifferentiated adaptive state, while inhibition of JUN-FAK-Src axis and BET could efficiently prevent the clonal evolution from quiescence to proliferation 159.

### Intratumoral support

#### Paracrine signals

*Tumor cell-cell interaction.* Inhibiting KRAS-RAF-MAPK signaling causes secretome remodeling 102, 198, 199. An elegant design using cell admixture of resistant and sensitive melanoma cells reveals that BRAFi resistant cells only proliferated in tumors with mixed cells, but not in homogenous tumors, suggesting that the paracrine signals from sensitive cells stimulate the outgrowth of resistant cells in response to treatment 198. BRAFi-induced *Fosl1* downregulation remodels the secretome in sensitive cells that stimulates resistant cells to grow, infiltrate and migrate partially via AKT pathway activation. Growth factors such as IGF1, EGF, ANGPTL7 and PDGFD are mediators of the intercellular interaction.

*Tumor-stroma cell interaction.* Tumor associated macrophages are not only sufficient but also essential for KRAS-independent tumor growth 102, providing the first evidence that immune cells play a critical role in regulating KRAS targeted therapy resistance. KRAS-depleted PDAC cells recruit macrophages via CCL2/7-CCR2 axis and reprogram them to an immature, M2-like state. Macrophages, in turn, served as the major source of TGFβ in TME that activated SMAD3/4-dependent canonical TGFβ signaling in tumor cells.

#### Matrix support

An interesting work investigating MEKi response in PDAC organoid reveals that, while cells in the interior layers underwent apoptosis, cells in the outer layer could sustain proliferation 200, The β1 integrin is required for cell-Matrigel interaction, depletion of which triggers cell death in both layers and overcomes MEKi resistance. In addition, interaction with fibroblasts desensitizes melanoma cells to BRAFi, while depletion of fibronectin in tumor cells suppresses NRG and HGF-mediated AKT activation 201. Moreover, proliferative MEKi resistant melanoma cells tend to colocalize with bundled collagen 202, implying that matrix interaction provides tumor cells with survival signals.

## Major challenges of KRAS targeted therapy and future perspectives

Great progresses in the past decade have changed the paradigm from "undruggable RAS" to “RAS is druggable”. Though G12Ci has shown promising results in clinical trials, development of inhibitors targeting other KRAS mutants remains challenging. As we have discussed above, other KRAS inhibitory methods have their own technical uncertainties, and thus only clinical trials will judge their feasibility. Another challenge to target KRAS is to prevent therapy resistance. Given that various molecular mechanisms and factors have been identified, significant knowledge gaps remain to be filled to overcome the common problem for targeted therapy. Here, we discuss six precedent research areas:

(1) While KRAS is frequently mutated in human cancers, the much higher tumor response rate to G12Ci in NSCLC patients versus CRC patients implies that the dependency on KRAS signaling pathway is distinct between various cancer types. It has been shown that activation of RTKs especially EGFR accounts for the reactivation of MAPK signaling pathway and the non-responsiveness of KRASi in CRC 119. Another possibility of the lack of responsiveness in CRC patients may be due to the mosaicism of wildtype and mutant KRAS in tumors that the continued growth of subclones with wildtype KRAS takes over the tumors under KRASi treatment. Mouse model studies revealed that conditional null alleles of *Apc* and *Trp53* are essential and sufficient to drive colonic tumorigenesis, while KRAS is only required for metastasis 203, 204. Inactivation of KRAS in autochthonous tumors transiently suppressed CRC growth followed by tumor recurrence. In addition, the gut microbiota is distinct in KRAS mutated and wildtype CRC groups 205, which may regulate tumor response to KRASi as well. Thus, understanding the difference between cancer types in responding to KRASi by comprehensive analysis of accumulated clinical data and employing genetic cancer models are crucial for resistance mechanism dissection and development of cancer type specific treatment approaches.

(2) Given that the genetic heterogeneity affects tumor response to KRAS targeted therapy, our knowledge on the roles of common cancer genetic alternations in regulating therapy resistance remains largely unknown. Early-stage clinical trial of adagrasib suggested that NSCLC patients harboring mutated *STK11* (aka *LKB1*), a recurrent tumor suppressor in 13% lung cancer patients, may respond better than wildtype ones (KRYSTAL-1 study), though none of *TP53*, *STK11* and *KEAP1* mutational status has a clear association with NSCLC patient response to sotorasib in another clinical trial 37. *USP21*, an oncogene amplified in 4.5% NSCLC patients and 3.3% PDAC patients (TCGA PanCancer Atlas), has been shown to promote pancreatic cancer cells to bypass the dependency of KRAS 186. In addition, depletion of tumor suppressor *SMAD4* prevents TGFβ-driven KRASi resistance in pancreatic cancer models 102. Taken together, comprehensive studies using functional genomics approaches, genetically engineered mouse models and patient-derived xenograft models are needed to understand the function of oncogenes, tumor suppressors and other frequently altered genes, miRNAs and lncRNAs in human cancers upon targeting KRAS and elucidate the molecular mechanisms leading to therapy hypersensitivity or refractoriness. The dependency of KRAS signaling in KRASi refractory tumor cells and how the residual tumor cells in a quiescent state survive, re-proliferate, and eventually form a relapsed tumor need to be determined for effective combination therapy.

(3) KRAS signaling inhibition remodels tumor microenvironment that can be exploited to enhance tumoricidal effect. Dual inhibition of MEK and CDK4/6 induces cell senescence in PDAC and remodels cell secretome that promotes tumor vascularization, which in turn enhances drug delivery and CD8+ T cell infiltration 199. As expected, chemotherapy drug gemcitabine caused significant tumor shrinkage in combination with MEK and CDK4/6 inhibitors, and blockage of tumor vascularization by VEGFR antibody neutralized the effect. Moreover, PD-1 blockade that leverages the infiltrated CD8+ T cells further suppressed tumor growth relative to dual inhibition of MEK and CDK4/6. Another example is G12Ci treatment in CRC mouse models 34, 206. KRASi treatment increases immune cell infiltration including CD8+ T cells, macrophages, and dendritic cells, and stimulates IFN and chemokine expression, providing a pro-inflammatory tumor microenvironment suitable for immune therapy combinations. Indeed, though monotherapy shows only moderate tumoricidal effect, PD-1 immune checkpoint blockade synergized with sotorasib or adagrasib to significantly prolong mouse survival. It is critical to delineate the intercellular crosstalk and identify the paracrine signals within tumor milieu under KRAS targeted therapy.

(4) Oncogene addiction is a process in which cancers dependent on one or several genes for maintenance and survival. Accumulated evidence suggests that tumor cells are prone to reactivate the addicted oncogene and corresponding signaling pathways to sustain tumor growth once it gets inhibited, providing the rationale to induce collateral lethality by targeting the vertical signaling pathway. ATP-competitive RAF kinase inhibitors effectively inhibit ERK signaling in BRAF mutant tumor cells, but they activate ERK signaling in tumor cells with wildtype BRAF or mutant RAS 207-212. The paradoxical effect is linked with conformational changes of RAF kinase domain when bound with inhibitors 209. Mutant BRAF as monomer activates ERK independent of RAS, but drug-inhibited BRAF transactivates drug-free protomer of CRAF homodimers (CRAF-CRAF) or heterodimers (CRAF-BRAF) to elevate MAPK signaling in a RAS-dependent manner 207-209. In addition, RAFi can relieve the negative feedback of Spry proteins on RTK-RAS signaling, followed by potentiation of mitogenic signaling from growth factors 213. Consistently, several growth factors such as EGF, HGF and NRG1 potentiality drive BRAFi resistance in BRAF V600E melanoma models, and combined inhibition of BRAF and MEK nearly completely blocked the rebound of ERK signaling 210, 213. Moreover, MEKi enhances the cytotoxicity of G12Ci sotorasib and impairs tumor growth 34. Taken together, vertical pathway activation is the major resistance mechanism to targeted therapy, which has already been extensively studied. The challenge is to identify the key node in KRAS signaling pathway to prevent activation of most surrogate factors and develop therapeutics with acceptable AEs.

(5) Inhibition of KRAS-MEK-ERK cascade causes increased autophagosome formation and mitochondrial stress in PDAC cells 162, 186, 188, 189, indicating the imbalance of metabolic homeostasis upon treatment. KRAS mutant PDAC cells have high demand of amino acids, thus scavenger pathways such as macropinocytosis and autophagy as well as *de novo* amino acid synthesis pathways are upregulated. The elevation of autophagy upon KRAS signaling inhibition compensates the downregulation of macropinocytosis and amino acid synthesis activity to salvage the cells, while autophagy inhibitors are efficient to enhance tumoricidal effect of KRAS signaling inhibitors. KRAS-depleted PDAC cells also switch from glycolysis to OXPHOS to fulfil their energy requirement, inhibition of which impairs cell survival. These studies imply a new direction to prevent resistance by exacerbating the metabolic imbalance induced by KRAS targeted therapy. Except for amino acid and energy homeostasis, whether other metabolites involving in key biological events such as epigenetics and lipid metabolism are deregulated or contributed to therapy resistance need to be determined by comprehensive metabolomics analysis.

(6) Complete response cases are all alike, every relapsed tumor is relapsed in its own way. The heterogeneity is the elephant in the room that we have to not only explore the possible common adaptive mechanisms of KRAS targeted therapy resistance but also think about how to manage tumor evolution. EMT and tumor associated macrophages are correlated with resistance to targeted therapy, chemotherapy, radiotherapy, and immunotherapy in various cancers, implying that they may be common drivers of therapy resistance. In addition, vertical pathway activation is another major resistance mechanism to targeted therapy as aforementioned above. Further studies need to be performed to dissect the molecular mechanism how EMT reprograms oncogene addiction, how tumor associated macrophages nourish tumor cells under KRAS targeted therapy, and how the vertical pathway is reactivated. On the other hand, given that treatment induced tumor evolution seems inevitable, elimination of the dormant cancer stem cells survived under oncogene targeted therapy may prevent tumor cell evolution and recurrence, which needs to be comprehensively explored in genetically engineered mouse models.

Collectively, there is a growing consensus that combination therapy rather than KRASi monotherapy is essential to achieve favorable clinical benefits. Approaches on different combinations, including vertical pathway inhibition, immune checkpoint blockade and chemotherapeutic regimens, are actively being investigated in clinical trials. It is noted that the various targeting KRAS methods have distinct working mechanisms, thus unique combination strategies may be required to prevent resistance. Alternatively, KRASi-induced reprogramming of tumor cell intrinsic dependency and TME remodeling provides opportunities to exploit the treatment-dependent cancer vulnerabilities. In summary, the central goal in the coming decade is to enhance tumoricidal effect of KRAS targeted therapy by leveraging the advantages of different therapeutics and finding the optimal combinatory approaches.

## Figures and Tables

**Table 1:** List of therapeutic approaches targeting KRAS-RAF-MEK in clinical trials and FDA-approved

Therapy	Direct target	Cancer type	Trial phase and combinations	Clinical trial ID#	Sponsor
AMG 510 (sotorasib)	KRAS G12C	KRAS^G12C^ mutant NSCLC	Ph3	NCT04303780	Amgen
		KRAS^G12C^ mutant advanced cancers	Ph1/2	NCT03600883	
			Ph1; with inhibitors targeting PD1, MEK, SHP2, pan-ErbB, PD-L1 or EGFR, or with chemotherapeutic regimen	NCT04185883	
			Ph1	NCT04380753	
MRTX849 (adagrasib)	KRAS G12C	KRAS^G12C^ mutant advanced cancers	Ph1/2; with TNO155 (SHP2 inhibitor)	NCT04330664	Mirati
			Ph1/2; monotherapy and with pembrolizumab, cetuximab or afatinib	NCT03785249	
JNJ-74699157	KRAS G12C	KRAS^G12C^ mutant advanced cancers	Ph1	NCT04006301	Janssen
LY3499446	KRAS G12C	KRAS^G12C^ mutant advanced cancers	Ph1/2; monotherapy and with abemaciclib, cetuximab, erlotinib or docetaxel	NCT04165031	Eli Lilly
iExosomes	KRAS G12D	KRAS^G12D^ mutant mPDAC	Ph1	NCT03608631	MDACC
V941 (mRNA-5671)	mutant KRAS	KRAS mutant mNSCLC, mCRC or mPDAC	Ph1; monotherapy and with pembrolizumab	NCT03948763	Merck
anti-KRAS G12D mTCR PBL	KRAS G12D	KRAS^G12D^ mutant cancers	Ph1/2	NCT03745326	NCI
Anti-KRAS G12V mTCR PBL	KRAS G12V	KRAS^G12V^ mutant cancers	Ph1/2	NCT03190941	NCI
BI 1701963	SOS1	KRAS mutant cancers	Ph1; monotherapy and with trametinib	NCT04111458	Boehringer Ingelheim
Rigosertib	RBD domain	KRAS mutant advanced NSCLC (first line treatment refractory)	Ph1/2; with nivolumab	NCT04263090	Onconova
Vemurafenib	BRAF V600E	BRAF^V600E^ mutant metastatic melanoma	Approved	/	Genentech
Dabrafenib	BRAF V600E	BRAF^V600E/K^ mutant metastatic melanoma	Approved; monotherapy and with trametinib	/	GlaxoSmithKline
Encorafenib	BRAF V600E	BRAF^V600E/K^ mutant metastatic melanoma and mCRC	Approved; with binimetinib (melanoma) or with cetuximab (CRC)	/	Novartis
LXH254	RAF and RAF dimer	MAPK pathway altered advanced cancers	Ph1	NCT02607813	Novartis
		metastatic melanoma	Ph2; with LTT462, trametinib or ribociclib	NCT04417621	
		KRAS or BRAF mutant NSCLC and NRAS mutant melanoma	Ph1; with LTT462, trametinib or ribociclib	NCT02974725	
PLX8394	BRAF dimer	BRAF mutant advanced cancers	Ph1/2	NCT02428712	Plexxikon
Lifirafenib (BGB-283)	BRAF V600E and EGFR	Advanced or treatment refractory cancers	Ph1/2; with PD-0325901	NCT03905148	BeiGene
Trametinib (GSK1120212)	MEK1/2	BRAF^V600E/K^ mutant metastatic melanoma	Approved; monotherapy and with dabrafenib	/	GlaxoSmithKline
Cobimetinib (GDC-0973)	MEK1	BRAF^V600E/K^ mutant metastatic melanoma	Approved; with vemurafenib	/	Genentech
Binimetinib (ARRY-162)	MEK	BRAF^V600E/K^ mutant metastatic melanoma	Approved; with encorafenib	/	Array
Mirdametinib (PD-0325901)	MEK1/2	KRAS mutant advanced NSCLC	Ph1/2; with dacomitinib	NCT02039336	Pfizer

Ph, Phase; mPDAC, mNSCLC and mCRC, metastatic PDAC, NSCLC and CRC.

**Table 2:** Adaptive resistance mechanisms to RAS-RAF-MEK signaling inhibition

Regulator	Role	Agent	Cancer type	Mechanism	References
AURKA	Pro	G12Ci	KRAS^G12C^ mutant NSCLC	Induces adaptive KRAS signaling reactivation	158
autophagy	Pro	G12Ci; MEKi; ERKi	KRAS mutant PDAC, NRAS mutant melanoma, BRAF mutant CRC	Pro-survival	162, 188, 189
AXL	Pro	MEKi	KRAS mutant PDAC	Activates PI3K/AKT/mTOR pathway	121
BCL-XL/ BCL-2	Pro	MEKi+PI3Ki	KRAS mutant NSCLC	Pro-survival	194
BET	Pro	dual MEKi and TBKi; MEKi; BRAFi	KRAS mutant NSCLC, CRC, TNBC, MM and PDAC; BRAF^V600E^ mutant melanoma	Required for treatment-induced chromatin remodeling	105, 106, 108, 109, 159
CCL2	Pro	KRASi	KRAS mutant PDAC	Recruits TGFβ-secreting M2-like macrophages to support KRAS independent tumor growth	102
CDK4/6	Pro	G12Ci	KRAS^G12C^ cancers	Required for tumor cell growth	33, 34, 118, 143
CDK7/12	Pro	BRAFi, MEKi	KRAS mutant NSCLC and gastric cancer, BRAF mutant melanoma	Required for treatment induced transcriptional and epigenetic remodeling	163
chemotherapy	Anti	MEKi+CDK4/6 i	KRAS mutant PDAC	Causes cell death	199
COT/TPL2	Pro	RAFi; MEKi	BRAF^V600E^ mutant melanoma	Activates MAPK independent of RAF	166
CRAF	Pro	BRAFi	BRAF mutant melanoma and CRC	Supports transactivation of RAS-MAPK signaling	138
ERK	Pro	RAFi, BRAFi+MEKi, BRAFi+EGFRi	BRAF mutant CRC, KRAS mutant PDAC	Promotes resistance	86, 145, 214
FAK	Pro	BRAFi and MEKi	BRAF^V600E^ mutant melanoma	Required for cell proliferation	159
FGFR	Pro	G12Ci; MEKi	KRAS mutant cancers	Promotes reactivation of MAPK signaling	118, 126
HAT1	Anti	dual BRAFi and MEKi	BRAF^V600E^ mutant melanoma	Loss of HAT1 upregulates MAPK via IGF1R	96
HDAC5	Pro	MEKi; KRASi	KRAS mutant PDAC	Suppresses Socs3 to reprogram chemokine expression	102
HDAC family	Pro	MEKi	PDAC, uveal melanoma and CRC	Required for treatment-induced upregulation of pAKT and YAP1	98-100
HER family	Pro	G12Ci; BRAFi; MEKi	KRAS or BRAF mutant CRC, thyroid cancer, NSCLC, LGSC and melanoma	Promotes reactivation of MAPK and AKT-mTOR pathways	33, 34, 113-120, 122, 123, 158, 215
HSP90	Pro	G12Ci; BRAFi; MEKi	RAS or RAF mutant MM and NSCLC	Amplifies MAPK signaling	143, 182, 183
IGF1R	Pro	G12Ci; BRAFi; MEKi	KRAS or BRAF mutant CRC, NSCLC, melanoma	Activates PI3K pathway	131-135
JUN	Pro	BRAFi and MEKi	BRAF^V600E^ mutant melanoma	Required for cell proliferation	159
MEK	Pro	G12Ci; BRAFi	BRAF^V600E^ mutant melanoma, KRAS^G12C^ mutant cancers	Required for cell growth	34, 210, 213
MET	Pro	BRAFi	BRAF^V600E^ mutant ATC	Promotes MAPK reactivation	139
NRAS	Pro	RAFi+EGFRi	BRAF^V600E^ mutant CRC	Promotes RAF dimerization and activates ERK	88
OXPHOS	Pro	KRASi	KRAS mutant PDAC	Adaptive energenic metabolism	162
P-TEFb complex	Pro	MEKi	TNBC	Upregulates RTKs by promoting *de novo* enhancer formation	107
PD-L1	Pro	MEKi+CDK4/6i	KRAS mutant PDAC	Causes exhaustion of infiltrated CD8+ T cells	199
PDGFRα	Pro	RAFi; MEKi	BRAF^V600E^ mutant melanoma, KRAS mutant PDAC	Activates JAK/STAT3	124, 125
PI3K-AKT-mTOR	Pro	G12Ci; BRAFi; MEKi	RAS or RAF mutant cancers	Supports drug resistant cell growth, infiltration and metastasis; supports drug sensitive cell survival	33, 34, 110, 121, 143, 161, 168, 170, 198, 201, 216
PP2A	Anti	MEKi	KRAS mutant NSCLC	Suppresses MAP3K2	157
SETD5	Pro	MEKi	KRAS mutant PDAC	Suppresses cytochrome P450 and glutathione metabolism pathways	101
SHOC2	Pro	RAFi; MEKi	KRAS mutant PDAC and NSCLC, BRAF mutant CRC	Required for growth factor-mediated RAS signaling activation	111, 138
SHP2	Pro	G12Ci; BRAFi; MEKi	BRAF mutant CRC, KRAS mutant cancers	Mediates RTK (e.g., EGFR)-induced RAS/MAPK reactivation	33, 34, 112, 118, 142, 143, 148-150, 158
Src family	Pro	BRAFi, MEKi	KRAS mutant cancers, BRAF^V600E^ melanoma	Promotes cancer cell survival and proliferation	159, 165
STAT3	Pro	MEKi	NSCLC	Pro-survival	172, 173
SUZ12	Anti	MEKi	MPNST	Amplifies Ras-driven transcription	1
TBK1	Pro	MEKi	NRAS mutant melanoma, KRAS mutant NSCLC	Activates STAT3 by inducing autocrine IL-6 and CCL5	154, 155
TGFβ	Pro	KRASi; MEKi	KRAS mutant NSCLC	Induces EMT and activates PI3K through FGFR1	102, 127
USP28	Anti	MEKi	BRAF^V600E^ melanoma	Stabilizes FBW7 to degrade BRAF	184
WNT5A	Pro	BRAFi	BRAF mutant melanoma	Activates PI3K/AKT	174
YAP1	Pro	KRASi; RAFi; MEKi; dual MEKi and EGFRi	BRAF or RAS mutant PDAC, NSCLC, melanoma, CRC and thyroid cancer	Activates cell cycle and DNA replication; regulates EMT; suppresses pro-apoptotic genes	178-181
ZEB1	Pro	MEKi	KRAS mutant NSCLC	Induces EMT and promotes MAPK-independent cell proliferation	196
β-catenin	Pro	MEKi	KRAS mutant CRC	Pro-survival	176
β1 integrin	Pro	MEKi	KRAS mutant PDAC	Pro-survival	200

Pro, pro-resistance; anti, anti-resistance; ATC, anaplastic thyroid cancer.

## Abbreviations

PDAC: pancreatic ductal adenocarcinoma
CRC: colorectal cancer
NSCLC: non-small cell lung cancer
GEM: genetically engineered mouse
GEFs: guanine nucleotide exchange-factors
GAPs: GTPase-activating proteins
RTKs: receptor tyrosine kinases
GPCRs: G-protein coupled receptors
TCGA: Cancer Genome Atlas
TME: tumor microenvironment
AEs: adverse events
G12Ci: KRAS G12C inhibitors
ORR: objective response rate
PFS: progression-free survival
SOC: standard of care
KRASi: KRAS inhibitors
TILs: tumor-infiltrated lymphocytes
TCR: T cell receptor
PBL: peripheral blood lymphocytes
mTCR: murine TCR
RBD: RAS-binding domain
MEKi: MEK inhibitors
RAFi: RAF inhibitor
HDAC: histone deacetylase
BET: Bromo and Extra Terminal Domain
BETi: BET inhibitors
TNBC: triple-negative breast cancer
MM: multiple myeloma
MPNSTs: malignant peripheral nerve sheath tumors
HGF: hepatocyte growth factor
PTP: protein-tyrosine phosphatase
SHP2i: SHP2 inhibition
PP1c: catalytic subunit of protein phosphatase 1
CSCs: cancer stem cells
EMT: epithelial-to-mesenchymal transition
PDK4: pyruvate dehydrogenase kinase 4
OXPHOS: oxidative phosphorylation
EMT-TFs: EMT transcription factors
Ph: Phase
mPDAC: mNSCLC and mCRC: metastatic PDAC: NSCLC and CRC
Pro: pro-resistance
anti: anti-resistance
ATC: anaplastic thyroid cancer

## References

[1] De Raedt T, Beert E, Pasmant E, Luscan A, Brems H, Ortonne N (2014). PRC2 loss amplifies Ras-driven transcription and confers sensitivity to BRD4-based therapies. Nature.

[2] Simanshu DK, Nissley DV, McCormick F (2017). RAS Proteins and Their Regulators in Human Disease. Cell.

[3] McGrath JP, Capon DJ, Goeddel DV, Levinson AD (1984). Comparative biochemical properties of normal and activated human ras p21 protein. Nature.

[4] Gibbs JB, Sigal IS, Poe M, Scolnick EM (1984). Intrinsic GTPase activity distinguishes normal and oncogenic ras p21 molecules. Proc Natl Acad Sci U S A.

[5] Sweet RW, Yokoyama S, Kamata T, Feramisco JR, Rosenberg M, Gross M (1984). The product of ras is a GTPase and the T24 oncogenic mutant is deficient in this activity. Nature.

[6] Scheffzek K, Ahmadian MR, Kabsch W, Wiesmuller L, Lautwein A, Schmitz F (1997). The Ras-RasGAP complex: structural basis for GTPase activation and its loss in oncogenic Ras mutants. Science.

[7] Gimple RC, Wang X (2019). RAS: Striking at the Core of the Oncogenic Circuitry. Front Oncol.

[8] Ambrogio C, Kohler J, Zhou ZW, Wang H, Paranal R, Li J (2018). KRAS Dimerization Impacts MEK Inhibitor Sensitivity and Oncogenic Activity of Mutant KRAS. Cell.

[9] Pupo E, Avanzato D, Middonti E, Bussolino F, Lanzetti L (2019). KRAS-Driven Metabolic Rewiring Reveals Novel Actionable Targets in Cancer. Front Oncol.

[10] Pylayeva-Gupta Y, Grabocka E, Bar-Sagi D (2011). RAS oncogenes: weaving a tumorigenic web. Nat Rev Cancer.

[11] Tape CJ, Ling S, Dimitriadi M, McMahon KM, Worboys JD, Leong HS (2016). Oncogenic KRAS Regulates Tumor Cell Signaling via Stromal Reciprocation. Cell.

[12] McAllister F, Bailey JM, Alsina J, Nirschl CJ, Sharma R, Fan H (2014). Oncogenic Kras activates a hematopoietic-to-epithelial IL-17 signaling axis in preinvasive pancreatic neoplasia. Cancer Cell.

[13] Dey P, Li J, Zhang J, Chaurasiya S, Strom A, Wang H (2020). Oncogenic KRAS-Driven Metabolic Reprogramming in Pancreatic Cancer Cells Utilizes Cytokines from the Tumor Microenvironment. Cancer Discov.

[14] Coelho MA, de Carne Trecesson S, Rana S, Zecchin D, Moore C, Molina-Arcas M (2017). Oncogenic RAS Signaling Promotes Tumor Immunoresistance by Stabilizing PD-L1 mRNA. Immunity.

[15] Zdanov S, Mandapathil M, Abu Eid R, Adamson-Fadeyi S, Wilson W, Qian J (2016). Mutant KRAS Conversion of Conventional T Cells into Regulatory T Cells. Cancer Immunol Res.

[16] Liao W, Overman MJ, Boutin AT, Shang X, Zhao D, Dey P (2019). KRAS-IRF2 Axis Drives Immune Suppression and Immune Therapy Resistance in Colorectal Cancer. Cancer Cell.

[17] Pylayeva-Gupta Y, Lee KE, Hajdu CH, Miller G, Bar-Sagi D (2012). Oncogenic Kras-induced GM-CSF production promotes the development of pancreatic neoplasia. Cancer Cell.

[18] Karnoub AE, Weinberg RA (2008). Ras oncogenes: split personalities. Nat Rev Mol Cell Biol.

[19] Dillon M, Lopez A, Lin E, Sales D, Perets R, Jain P (2021). Progress on Ras/MAPK Signaling Research and Targeting in Blood and Solid Cancers. Cancers (Basel).

[20] Braicu C, Buse M, Busuioc C, Drula R, Gulei D, Raduly L (2019). A Comprehensive Review on MAPK: A Promising Therapeutic Target in Cancer. Cancers (Basel).

[21] Leevers SJ, Paterson HF, Marshall CJ (1994). Requirement for Ras in Raf activation is overcome by targeting Raf to the plasma membrane. Nature.

[22] Stokoe D, Macdonald SG, Cadwallader K, Symons M, Hancock JF (1994). Activation of Raf as a result of recruitment to the plasma membrane. Science.

[23] Lavoie H, Gagnon J, Therrien M (2020). ERK signalling: a master regulator of cell behaviour, life and fate. Nat Rev Mol Cell Biol.

[24] Sanchez-Vega F, Mina M, Armenia J, Chatila WK, Luna A, La KC (2018). Oncogenic Signaling Pathways in The Cancer Genome Atlas. Cell.

[25] John J, Rensland H, Schlichting I, Vetter I, Borasio GD, Goody RS (1993). Kinetic and structural analysis of the Mg(2+)-binding site of the guanine nucleotide-binding protein p21H-ras. The Journal of biological chemistry.

[26] Noonan T, Brown N, Dudycz L, Wright G (1991). Interaction of GTP derivatives with cellular and oncogenic ras-p21 proteins. J Med Chem.

[27] Ostrem JM, Shokat KM (2016). Direct small-molecule inhibitors of KRAS: from structural insights to mechanism-based design. Nat Rev Drug Discov.

[28] James GL, Goldstein JL, Brown MS (1995). Polylysine and CVIM sequences of K-RasB dictate specificity of prenylation and confer resistance to benzodiazepine peptidomimetic in vitro. The Journal of biological chemistry.

[29] Ostrem JM, Peters U, Sos ML, Wells JA, Shokat KM (2013). K-Ras(G12C) inhibitors allosterically control GTP affinity and effector interactions. Nature.

[30] Patricelli MP, Janes MR, Li LS, Hansen R, Peters U, Kessler LV (2016). Selective Inhibition of Oncogenic KRAS Output with Small Molecules Targeting the Inactive State. Cancer Discov.

[31] Zeng M, Lu J, Li L, Feru F, Quan C, Gero TW (2017). Potent and Selective Covalent Quinazoline Inhibitors of KRAS G12C. Cell Chem Biol.

[32] Janes MR, Zhang J, Li LS, Hansen R, Peters U, Guo X (2018). Targeting KRAS Mutant Cancers with a Covalent G12C-Specific Inhibitor. Cell.

[33] Hallin J, Engstrom LD, Hargis L, Calinisan A, Aranda R, Briere DM (2020). The KRAS(G12C) Inhibitor MRTX849 Provides Insight toward Therapeutic Susceptibility of KRAS-Mutant Cancers in Mouse Models and Patients. Cancer Discov.

[34] Canon J, Rex K, Saiki AY, Mohr C, Cooke K, Bagal D (2019). The clinical KRAS(G12C) inhibitor AMG 510 drives anti-tumour immunity. Nature.

[35] Lito P, Solomon M, Li LS, Hansen R, Rosen N (2016). Allele-specific inhibitors inactivate mutant KRAS G12C by a trapping mechanism. Science.

[36] Hong DS, Fakih MG, Strickler JH, Desai J, Durm GA, Shapiro GI (2020). KRAS(G12C) Inhibition with Sotorasib in Advanced Solid Tumors. N Engl J Med.

[37] Skoulidis F, Li BT, Dy GK, Price TJ, Falchook GS, Wolf J (2021). Sotorasib for Lung Cancers with KRAS p.G12C Mutation. N Engl J Med.

[38] Kamerkar S, LeBleu VS, Sugimoto H, Yang S, Ruivo CF, Melo SA (2017). Exosomes facilitate therapeutic targeting of oncogenic KRAS in pancreatic cancer. Nature.

[39] Mendt M, Kamerkar S, Sugimoto H, McAndrews KM, Wu CC, Gagea M (2018). Generation and testing of clinical-grade exosomes for pancreatic cancer. JCI Insight.

[40] Pardi N, Hogan MJ, Porter FW, Weissman D (2018). mRNA vaccines - a new era in vaccinology. Nat Rev Drug Discov.

[41] Yamamoto K, Venida A, Yano J, Biancur DE, Kakiuchi M, Gupta S (2020). Autophagy promotes immune evasion of pancreatic cancer by degrading MHC-I. Nature.

[42] Brea EJ, Oh CY, Manchado E, Budhu S, Gejman RS, Mo G (2016). Kinase Regulation of Human MHC Class I Molecule Expression on Cancer Cells. Cancer Immunol Res.

[43] El-Jawhari JJ, El-Sherbiny YM, Scott GB, Morgan RS, Prestwich R, Bowles PA (2014). Blocking oncogenic RAS enhances tumour cell surface MHC class I expression but does not alter susceptibility to cytotoxic lymphocytes. Mol Immunol.

[44] Rosenberg SA, Yang JC, Sherry RM, Kammula US, Hughes MS, Phan GQ (2011). Durable complete responses in heavily pretreated patients with metastatic melanoma using T-cell transfer immunotherapy. Clin Cancer Res.

[45] Tran E, Turcotte S, Gros A, Robbins PF, Lu YC, Dudley ME (2014). Cancer immunotherapy based on mutation-specific CD4+ T cells in a patient with epithelial cancer. Science.

[46] Kochenderfer JN, Dudley ME, Feldman SA, Wilson WH, Spaner DE, Maric I (2012). B-cell depletion and remissions of malignancy along with cytokine-associated toxicity in a clinical trial of anti-CD19 chimeric-antigen-receptor-transduced T cells. Blood.

[47] Robbins PF, Kassim SH, Tran TL, Crystal JS, Morgan RA, Feldman SA (2015). A pilot trial using lymphocytes genetically engineered with an NY-ESO-1-reactive T-cell receptor: long-term follow-up and correlates with response. Clin Cancer Res.

[48] Wang QJ, Yu Z, Griffith K, Hanada K, Restifo NP, Yang JC (2016). Identification of T-cell Receptors Targeting KRAS-Mutated Human Tumors. Cancer Immunol Res.

[49] Tran E, Robbins PF, Lu YC, Prickett TD, Gartner JJ, Jia L (2016). T-Cell Transfer Therapy Targeting Mutant KRAS in Cancer. N Engl J Med.

[50] Sim MJW, Lu J, Spencer M, Hopkins F, Tran E, Rosenberg SA (2020). High-affinity oligoclonal TCRs define effective adoptive T cell therapy targeting mutant KRAS-G12D. Proc Natl Acad Sci U S A.

[51] Rech AJ, Vonderheide RH (2017). T-Cell Transfer Therapy Targeting Mutant KRAS. N Engl J Med.

[52] Shin SM, Kim JS, Park SW, Jun SY, Kweon HJ, Choi DK (2020). Direct targeting of oncogenic RAS mutants with a tumor-specific cytosol-penetrating antibody inhibits RAS mutant-driven tumor growth. Sci Adv.

[53] Shin SM, Choi DK, Jung K, Bae J, Kim JS, Park SW (2017). Antibody targeting intracellular oncogenic Ras mutants exerts anti-tumour effects after systemic administration. Nat Commun.

[54] Athuluri-Divakar SK, Vasquez-Del Carpio R, Dutta K, Baker SJ, Cosenza SC, Basu I (2016). A Small Molecule RAS-Mimetic Disrupts RAS Association with Effector Proteins to Block Signaling. Cell.

[55] Kopetz S, Grothey A, Yaeger R, Van Cutsem E, Desai J, Yoshino T (2019). Encorafenib, Binimetinib, and Cetuximab in BRAF V600E-Mutated Colorectal Cancer. N Engl J Med.

[56] Dummer R, Ascierto PA, Gogas HJ, Arance A, Mandala M, Liszkay G (2018). Overall survival in patients with BRAF-mutant melanoma receiving encorafenib plus binimetinib versus vemurafenib or encorafenib (COLUMBUS): a multicentre, open-label, randomised, phase 3 trial. Lancet Oncol.

[57] Chapman PB, Hauschild A, Robert C, Haanen JB, Ascierto P, Larkin J (2011). Improved survival with vemurafenib in melanoma with BRAF V600E mutation. N Engl J Med.

[58] Robert C, Grob JJ, Stroyakovskiy D, Karaszewska B, Hauschild A, Levchenko E (2019). Five-Year Outcomes with Dabrafenib plus Trametinib in Metastatic Melanoma. N Engl J Med.

[59] Peng SB, Henry JR, Kaufman MD, Lu WP, Smith BD, Vogeti S (2015). Inhibition of RAF Isoforms and Active Dimers by LY3009120 Leads to Anti-tumor Activities in RAS or BRAF Mutant Cancers. Cancer Cell.

[60] Tang Z, Yuan X, Du R, Cheung SH, Zhang G, Wei J (2015). BGB-283, a Novel RAF Kinase and EGFR Inhibitor, Displays Potent Antitumor Activity in BRAF-Mutated Colorectal Cancers. Mol Cancer Ther.

[61] Zhang C, Spevak W, Zhang Y, Burton EA, Ma Y, Habets G (2015). RAF inhibitors that evade paradoxical MAPK pathway activation. Nature.

[62] Desai J, Gan H, Barrow C, Jameson M, Atkinson V, Haydon A (2020). Phase I, Open-Label, Dose-Escalation/Dose-Expansion Study of Lifirafenib (BGB-283), an RAF Family Kinase Inhibitor, in Patients With Solid Tumors. J Clin Oncol.

[63] Janku F, Iyer G, Spreafico A, Yamamoto N, Bang Y-J, Elez E (2018). A phase I study of LXH254 in patients (pts) with advanced solid tumors harboring MAPK pathway alterations. Journal of Clinical Oncology.

[64] Sullivan RJ, Hollebecque A, Flaherty KT, Shapiro GI, Rodon Ahnert J, Millward MJ (2020). A Phase I Study of LY3009120, a Pan-RAF Inhibitor, in Patients with Advanced or Metastatic Cancer. Mol Cancer Ther.

[65] Janku F, Vaishampayan U, Khemka V, Bhatty M, Zhang C, Hsu HH (2018). Abstract B176: Results of a phase I study of PLX8394, a next-generation BRAF inhibitor, in refractory solid tumors. Molecular Cancer Therapeutics.

[66] Cheng Y, Tian H (2017). Current Development Status of MEK Inhibitors. Molecules.

[67] Hatzivassiliou G, Haling JR, Chen H, Song K, Price S, Heald R (2013). Mechanism of MEK inhibition determines efficacy in mutant KRAS- versus BRAF-driven cancers. Nature.

[68] Lito P, Saborowski A, Yue J, Solomon M, Joseph E, Gadal S (2014). Disruption of CRAF-mediated MEK activation is required for effective MEK inhibition in KRAS mutant tumors. Cancer Cell.

[69] Koga T, Suda K, Fujino T, Ohara S, Hamada A, Nishino M (2021). KRAS Secondary Mutations That Confer Acquired Resistance to KRAS G12C Inhibitors, Sotorasib and Adagrasib, and Overcoming Strategies: Insights From In Vitro Experiments. J Thorac Oncol.

[70] Tanaka N, Lin JJ, Li C, Ryan MB, Zhang J, Kiedrowski LA (2021). Clinical Acquired Resistance to KRAS(G12C) Inhibition through a Novel KRAS Switch-II Pocket Mutation and Polyclonal Alterations Converging on RAS-MAPK Reactivation. Cancer Discov.

[71] Awad MM, Liu S, Rybkin, II, Arbour KC, Dilly J, Zhu VW (2021). Acquired Resistance to KRAS(G12C) Inhibition in Cancer. N Engl J Med.

[72] Zhao Y, Murciano-Goroff YR, Xue JY, Ang A, Lucas J, Mai TT (2021). Diverse alterations associated with resistance to KRAS(G12C) inhibition. Nature.

[73] Emery CM, Vijayendran KG, Zipser MC, Sawyer AM, Niu L, Kim JJ (2009). MEK1 mutations confer resistance to MEK and B-RAF inhibition. Proc Natl Acad Sci U S A.

[74] Gao Y, Maria A, Na N, da Cruz Paula A, Gorelick AN, Hechtman JF (2019). V211D Mutation in MEK1 Causes Resistance to MEK Inhibitors in Colon Cancer. Cancer Discov.

[75] Wagle N, Van Allen EM, Treacy DJ, Frederick DT, Cooper ZA, Taylor-Weiner A (2014). MAP kinase pathway alterations in BRAF-mutant melanoma patients with acquired resistance to combined RAF/MEK inhibition. Cancer Discov.

[76] Poulikakos PI, Persaud Y, Janakiraman M, Kong X, Ng C, Moriceau G (2011). RAF inhibitor resistance is mediated by dimerization of aberrantly spliced BRAF(V600E). Nature.

[77] Hartsough EJ, Basile KJ, Aplin AE (2014). Beneficial effects of RAF inhibitor in mutant BRAF splice variant-expressing melanoma. Mol Cancer Res.

[78] Johnson DB, Childress MA, Chalmers ZR, Frampton GM, Ali SM, Rubinstein SM (2018). BRAF internal deletions and resistance to BRAF/MEK inhibitor therapy. Pigment Cell Melanoma Res.

[79] Vido MJ, Le K, Hartsough EJ, Aplin AE (2018). BRAF Splice Variant Resistance to RAF Inhibitor Requires Enhanced MEK Association. Cell Rep.

[80] Shi H, Hugo W, Kong X, Hong A, Koya RC, Moriceau G (2014). Acquired resistance and clonal evolution in melanoma during BRAF inhibitor therapy. Cancer Discov.

[81] Yuan J, Ng WH, Tian Z, Yap J, Baccarini M, Chen Z (2018). Activating mutations in MEK1 enhance homodimerization and promote tumorigenesis. Sci Signal.

[82] Yap J, Deepak R, Tian Z, Ng WH, Goh KC, Foo A (2021). The stability of R-spine defines RAF inhibitor resistance: A comprehensive analysis of oncogenic BRAF mutants with in-frame insertion of alphaC-beta4 loop. Sci Adv.

[83] Shi H, Moriceau G, Kong X, Lee MK, Lee H, Koya RC (2012). Melanoma whole-exome sequencing identifies (V600E)B-RAF amplification-mediated acquired B-RAF inhibitor resistance. Nat Commun.

[84] Kemper K, Krijgsman O, Cornelissen-Steijger P, Shahrabi A, Weeber F, Song JY (2015). Intra- and inter-tumor heterogeneity in a vemurafenib-resistant melanoma patient and derived xenografts. EMBO Mol Med.

[85] Moriceau G, Hugo W, Hong A, Shi H, Kong X, Yu CC (2015). Tunable-combinatorial mechanisms of acquired resistance limit the efficacy of BRAF/MEK cotargeting but result in melanoma drug addiction. Cancer Cell.

[86] Ahronian LG, Sennott EM, Van Allen EM, Wagle N, Kwak EL, Faris JE (2015). Clinical Acquired Resistance to RAF Inhibitor Combinations in BRAF-Mutant Colorectal Cancer through MAPK Pathway Alterations. Cancer Discov.

[87] Oddo D, Sennott EM, Barault L, Valtorta E, Arena S, Cassingena A (2016). Molecular Landscape of Acquired Resistance to Targeted Therapy Combinations in BRAF-Mutant Colorectal Cancer. Cancer Res.

[88] Yaeger R, Yao Z, Hyman DM, Hechtman JF, Vakiani E, Zhao H (2017). Mechanisms of Acquired Resistance to BRAF V600E Inhibition in Colon Cancers Converge on RAF Dimerization and Are Sensitive to Its Inhibition. Cancer Res.

[89] Corcoran RB, Dias-Santagata D, Bergethon K, Iafrate AJ, Settleman J, Engelman JA (2010). BRAF gene amplification can promote acquired resistance to MEK inhibitors in cancer cells harboring the BRAF V600E mutation. Sci Signal.

[90] Little AS, Balmanno K, Sale MJ, Newman S, Dry JR, Hampson M (2011). Amplification of the driving oncogene, KRAS or BRAF, underpins acquired resistance to MEK1/2 inhibitors in colorectal cancer cells. Sci Signal.

[91] Hatzivassiliou G, Liu B, O'Brien C, Spoerke JM, Hoeflich KP, Haverty PM (2012). ERK inhibition overcomes acquired resistance to MEK inhibitors. Mol Cancer Ther.

[92] Zhang J, Liu M, Liu W, Wang W (2019). Ras-ERK1/2 signalling promotes the development of osteosarcoma through regulation of H4K12ac through HAT1. Artif Cells Nanomed Biotechnol.

[93] Han N, Shi L, Guo Q, Sun W, Yu Y, Yang L (2017). HAT1 induces lung cancer cell apoptosis via up regulating Fas. Oncotarget.

[94] Fan P, Zhao J, Meng Z, Wu H, Wang B, Wu H (2019). Overexpressed histone acetyltransferase 1 regulates cancer immunity by increasing programmed death-ligand 1 expression in pancreatic cancer. J Exp Clin Cancer Res.

[95] Gruber JJ, Geller B, Lipchik AM, Chen J, Salahudeen AA, Ram AN (2019). HAT1 Coordinates Histone Production and Acetylation via H4 Promoter Binding. Mol Cell.

[96] Bugide S, Parajuli KR, Chava S, Pattanayak R, Manna DLD, Shrestha D (2020). Loss of HAT1 expression confers BRAFV600E inhibitor resistance to melanoma cells by activating MAPK signaling via IGF1R. Oncogenesis.

[97] Mazur PK, Reynoird N, Khatri P, Jansen PW, Wilkinson AW, Liu S (2014). SMYD3 links lysine methylation of MAP3K2 to Ras-driven cancer. Nature.

[98] Carson R, Celtikci B, Fenning C, Javadi A, Crawford N, Carbonell LP (2015). HDAC Inhibition Overcomes Acute Resistance to MEK Inhibition in BRAF-Mutant Colorectal Cancer by Downregulation of c-FLIPL. Clin Cancer Res.

[99] Chao MW, Chang LH, Tu HJ, Chang CD, Lai MJ, Chen YY (2019). Combination treatment strategy for pancreatic cancer involving the novel HDAC inhibitor MPT0E028 with a MEK inhibitor beyond K-Ras status. Clin Epigenetics.

[100] Faiao-Flores F, Emmons MF, Durante MA, Kinose F, Saha B, Fang B (2019). HDAC Inhibition Enhances the In Vivo Efficacy of MEK Inhibitor Therapy in Uveal Melanoma. Clin Cancer Res.

[101] Wang Z, Hausmann S, Lyu R, Li TM, Lofgren SM, Flores NM (2020). SETD5-Coordinated Chromatin Reprogramming Regulates Adaptive Resistance to Targeted Pancreatic Cancer Therapy. Cancer Cell.

[102] Hou P, Kapoor A, Zhang Q, Li J, Wu CJ, Li J (2020). Tumor Microenvironment Remodeling Enables Bypass of Oncogenic KRAS Dependency in Pancreatic Cancer. Cancer Discov.

[103] Guerriero JL, Sotayo A, Ponichtera HE, Castrillon JA, Pourzia AL, Schad S (2017). Class IIa HDAC inhibition reduces breast tumours and metastases through anti-tumour macrophages. Nature.

[104] Shi J, Vakoc CR (2014). The mechanisms behind the therapeutic activity of BET bromodomain inhibition. Mol Cell.

[105] Wyce A, Matteo JJ, Foley SW, Felitsky DJ, Rajapurkar SR, Zhang XP (2018). MEK inhibitors overcome resistance to BET inhibition across a number of solid and hematologic cancers. Oncogenesis.

[106] Wagner S, Vlachogiannis G, De Haven Brandon A, Valenti M, Box G, Jenkins L (2019). Suppression of interferon gene expression overcomes resistance to MEK inhibition in KRAS-mutant colorectal cancer. Oncogene.

[107] Zawistowski JS, Bevill SM, Goulet DR, Stuhlmiller TJ, Beltran AS, Olivares-Quintero JF (2017). Enhancer Remodeling during Adaptive Bypass to MEK Inhibition Is Attenuated by Pharmacologic Targeting of the P-TEFb Complex. Cancer Discov.

[108] Kitajima S, Asahina H, Chen T, Guo S, Quiceno LG, Cavanaugh JD (2018). Overcoming Resistance to Dual Innate Immune and MEK Inhibition Downstream of KRAS. Cancer Cell.

[109] Guerra SL, Maertens O, Kuzmickas R, De Raedt T, Adeyemi RO, Guild CJ (2020). A Deregulated HOX Gene Axis Confers an Epigenetic Vulnerability in KRAS-Mutant Lung Cancers. Cancer Cell.

[110] Misale S, Fatherree JP, Cortez E, Li C, Bilton S, Timonina D (2019). KRAS G12C NSCLC Models Are Sensitive to Direct Targeting of KRAS in Combination with PI3K Inhibition. Clin Cancer Res.

[111] Sulahian R, Kwon JJ, Walsh KH, Pailler E, Bosse TL, Thaker M (2019). Synthetic Lethal Interaction of SHOC2 Depletion with MEK Inhibition in RAS-Driven Cancers. Cell Rep.

[112] Ryan MB, Fece de la Cruz F, Phat S, Myers DT, Wong E, Shahzade HA (2020). Vertical Pathway Inhibition Overcomes Adaptive Feedback Resistance to KRAS(G12C) Inhibition. Clin Cancer Res.

[113] Prahallad A, Sun C, Huang S, Di Nicolantonio F, Salazar R, Zecchin D (2012). Unresponsiveness of colon cancer to BRAF(V600E) inhibition through feedback activation of EGFR. Nature.

[114] Okimoto RA, Lin L, Olivas V, Chan E, Markegard E, Rymar A (2016). Preclinical efficacy of a RAF inhibitor that evades paradoxical MAPK pathway activation in protein kinase BRAF-mutant lung cancer. Proc Natl Acad Sci U S A.

[115] Fernandez ML, Dawson A, Hoenisch J, Kim H, Bamford S, Salamanca C (2019). Markers of MEK inhibitor resistance in low-grade serous ovarian cancer: EGFR is a potential therapeutic target. Cancer Cell Int.

[116] Corcoran RB, Ebi H, Turke AB, Coffee EM, Nishino M, Cogdill AP (2012). EGFR-mediated re-activation of MAPK signaling contributes to insensitivity of BRAF mutant colorectal cancers to RAF inhibition with vemurafenib. Cancer Discov.

[117] Girotti MR, Pedersen M, Sanchez-Laorden B, Viros A, Turajlic S, Niculescu-Duvaz D (2013). Inhibiting EGF receptor or SRC family kinase signaling overcomes BRAF inhibitor resistance in melanoma. Cancer Discov.

[118] Lou K, Steri V, Ge AY, Hwang YC, Yogodzinski CH, Shkedi AR (2019). KRAS(G12C) inhibition produces a driver-limited state revealing collateral dependencies. Sci Signal.

[119] Amodio V, Yaeger R, Arcella P, Cancelliere C, Lamba S, Lorenzato A (2020). EGFR blockade reverts resistance to KRAS G12C inhibition in colorectal cancer. Cancer Discov.

[120] McFall T, Trogdon M, Sisk-Hackworth L, Stites EC Inhibition of both mutant and wild-type RAS-GTP in KRAS G12C colorectal cancer through cotreatment with G12C and EGFR inhibitors. bioRxiv. 2019: 845263.

[121] Pettazzoni P, Viale A, Shah P, Carugo A, Ying H, Wang H (2015). Genetic events that limit the efficacy of MEK and RTK inhibitor therapies in a mouse model of KRAS-driven pancreatic cancer. Cancer Res.

[122] Montero-Conde C, Ruiz-Llorente S, Dominguez JM, Knauf JA, Viale A, Sherman EJ (2013). Relief of feedback inhibition of HER3 transcription by RAF and MEK inhibitors attenuates their antitumor effects in BRAF-mutant thyroid carcinomas. Cancer Discov.

[123] Sun C, Hobor S, Bertotti A, Zecchin D, Huang S, Galimi F (2014). Intrinsic resistance to MEK inhibition in KRAS mutant lung and colon cancer through transcriptional induction of ERBB3. Cell Rep.

[124] Nazarian R, Shi H, Wang Q, Kong X, Koya RC, Lee H (2010). Melanomas acquire resistance to B-RAF(V600E) inhibition by RTK or N-RAS upregulation. Nature.

[125] Sahu N, Chan E, Chu F, Pham T, Koeppen H, Forrest W (2017). Cotargeting of MEK and PDGFR/STAT3 Pathways to Treat Pancreatic Ductal Adenocarcinoma. Mol Cancer Ther.

[126] Manchado E, Weissmueller S, Morris JPt, Chen CC, Wullenkord R, Lujambio A (2016). A combinatorial strategy for treating KRAS-mutant lung cancer. Nature.

[127] Kitai H, Ebi H, Tomida S, Floros KV, Kotani H, Adachi Y (2016). Epithelial-to-Mesenchymal Transition Defines Feedback Activation of Receptor Tyrosine Kinase Signaling Induced by MEK Inhibition in KRAS-Mutant Lung Cancer. Cancer Discov.

[128] Warshamana-Greene GS, Litz J, Buchdunger E, Garcia-Echeverria C, Hofmann F, Krystal GW (2005). The insulin-like growth factor-I receptor kinase inhibitor, NVP-ADW742, sensitizes small cell lung cancer cell lines to the effects of chemotherapy. Clin Cancer Res.

[129] Valenciano A, Henriquez-Hernandez LA, Moreno M, Lloret M, Lara PC (2012). Role of IGF-1 receptor in radiation response. Transl Oncol.

[130] Jones HE, Goddard L, Gee JM, Hiscox S, Rubini M, Barrow D (2004). Insulin-like growth factor-I receptor signalling and acquired resistance to gefitinib (ZD1839; Iressa) in human breast and prostate cancer cells. Endocr Relat Cancer.

[131] Villanueva J, Vultur A, Lee JT, Somasundaram R, Fukunaga-Kalabis M, Cipolla AK (2010). Acquired resistance to BRAF inhibitors mediated by a RAF kinase switch in melanoma can be overcome by cotargeting MEK and IGF-1R/PI3K. Cancer Cell.

[132] Flanigan SA, Pitts TM, Newton TP, Kulikowski GN, Tan AC, McManus MC (2013). Overcoming IGF1R/IR resistance through inhibition of MEK signaling in colorectal cancer models. Clin Cancer Res.

[133] Molina-Arcas M, Hancock DC, Sheridan C, Kumar MS, Downward J (2013). Coordinate direct input of both KRAS and IGF1 receptor to activation of PI3 kinase in KRAS-mutant lung cancer. Cancer Discov.

[134] Molina-Arcas M, Moore C, Rana S, van Maldegem F, Mugarza E, Romero-Clavijo P (2019). Development of combination therapies to maximize the impact of KRAS-G12C inhibitors in lung cancer. Sci Transl Med.

[135] Ebi H, Corcoran RB, Singh A, Chen Z, Song Y, Lifshits E (2011). Receptor tyrosine kinases exert dominant control over PI3K signaling in human KRAS mutant colorectal cancers. J Clin Invest.

[136] Mo HN, Liu P (2017). Targeting MET in cancer therapy. Chronic Dis Transl Med.

[137] Engelman JA, Zejnullahu K, Mitsudomi T, Song Y, Hyland C, Park JO (2007). MET amplification leads to gefitinib resistance in lung cancer by activating ERBB3 signaling. Science.

[138] Whittaker SR, Cowley GS, Wagner S, Luo F, Root DE, Garraway LA (2015). Combined Pan-RAF and MEK Inhibition Overcomes Multiple Resistance Mechanisms to Selective RAF Inhibitors. Mol Cancer Ther.

[139] Knauf JA, Luckett KA, Chen KY, Voza F, Socci ND, Ghossein R (2018). Hgf/Met activation mediates resistance to BRAF inhibition in murine anaplastic thyroid cancers. J Clin Invest.

[140] Ostman A, Hellberg C, Bohmer FD (2006). Protein-tyrosine phosphatases and cancer. Nat Rev Cancer.

[141] Nichols RJ, Haderk F, Stahlhut C, Schulze CJ, Hemmati G, Wildes D (2018). RAS nucleotide cycling underlies the SHP2 phosphatase dependence of mutant BRAF-, NF1- and RAS-driven cancers. Nat Cell Biol.

[142] Fedele C, Li S, Teng KW, Foster C, Peng D, Ran H (2020). SHP2 Inhibition Abrogates Adaptive Resistance to KRAS<sup>G12C</sup>-Inhibition and Remodels the Tumor Microenvironment of <em>KRAS</em>-Mutant Tumors. bioRxiv. 2020.

[143] Santana-Codina N, Chandhoke AS, Yu Q, Malachowska B, Kuljanin M, Gikandi A (2020). Defining and Targeting Adaptations to Oncogenic KRAS(G12C) Inhibition Using Quantitative Temporal Proteomics. Cell Rep.

[144] Fedele C, Li S, Teng KW, Foster CJR, Peng D, Ran H (2021). SHP2 inhibition diminishes KRASG12C cycling and promotes tumor microenvironment remodeling. J Exp Med.

[145] Ozkan-Dagliyan I, Diehl JN, George SD, Schaefer A, Papke B, Klotz-Noack K (2020). Low-Dose Vertical Inhibition of the RAF-MEK-ERK Cascade Causes Apoptotic Death of KRAS Mutant Cancers. Cell Rep.

[146] Adachi Y, Ito K, Hayashi Y, Kimura R, Tan TZ, Yamaguchi R (2020). Epithelial-to-Mesenchymal Transition is a Cause of Both Intrinsic and Acquired Resistance to KRAS G12C Inhibitor in KRAS G12C-Mutant Non-Small Cell Lung Cancer. Clin Cancer Res.

[147] Chen YN, LaMarche MJ, Chan HM, Fekkes P, Garcia-Fortanet J, Acker MG (2016). Allosteric inhibition of SHP2 phosphatase inhibits cancers driven by receptor tyrosine kinases. Nature.

[148] Prahallad A, Heynen GJ, Germano G, Willems SM, Evers B, Vecchione L (2015). PTPN11 Is a Central Node in Intrinsic and Acquired Resistance to Targeted Cancer Drugs. Cell Rep.

[149] Ruess DA, Heynen GJ, Ciecielski KJ, Ai J, Berninger A, Kabacaoglu D (2018). Mutant KRAS-driven cancers depend on PTPN11/SHP2 phosphatase. Nat Med.

[150] Mainardi S, Mulero-Sanchez A, Prahallad A, Germano G, Bosma A, Krimpenfort P (2018). SHP2 is required for growth of KRAS-mutant non-small-cell lung cancer in vivo. Nat Med.

[151] Rodriguez-Viciana P, Oses-Prieto J, Burlingame A, Fried M, McCormick F (2006). A phosphatase holoenzyme comprised of Shoc2/Sur8 and the catalytic subunit of PP1 functions as an M-Ras effector to modulate Raf activity. Mol Cell.

[152] Chien Y, Kim S, Bumeister R, Loo YM, Kwon SW, Johnson CL (2006). RalB GTPase-mediated activation of the IkappaB family kinase TBK1 couples innate immune signaling to tumor cell survival. Cell.

[153] Neel NF, Martin TD, Stratford JK, Zand TP, Reiner DJ, Der CJ (2011). The RalGEF-Ral Effector Signaling Network: The Road Less Traveled for Anti-Ras Drug Discovery. Genes Cancer.

[154] Zhu Z, Aref AR, Cohoon TJ, Barbie TU, Imamura Y, Yang S (2014). Inhibition of KRAS-driven tumorigenicity by interruption of an autocrine cytokine circuit. Cancer Discov.

[155] Vu HL, Aplin AE (2014). Targeting TBK1 inhibits migration and resistance to MEK inhibitors in mutant NRAS melanoma. Mol Cancer Res.

[156] Rangarajan A, Hong SJ, Gifford A, Weinberg RA (2004). Species- and cell type-specific requirements for cellular transformation. Cancer Cell.

[157] Kauko O, O'Connor CM, Kulesskiy E, Sangodkar J, Aakula A, Izadmehr S (2018). PP2A inhibition is a druggable MEK inhibitor resistance mechanism in KRAS-mutant lung cancer cells. Sci Transl Med.

[158] Xue JY, Zhao Y, Aronowitz J, Mai TT, Vides A, Qeriqi B (2020). Rapid non-uniform adaptation to conformation-specific KRAS(G12C) inhibition. Nature.

[159] Fallahi-Sichani M, Becker V, Izar B, Baker GJ, Lin JR, Boswell SA (2017). Adaptive resistance of melanoma cells to RAF inhibition via reversible induction of a slowly dividing de-differentiated state. Mol Syst Biol.

[160] Martinelli E, Troiani T, D'Aiuto E, Morgillo F, Vitagliano D, Capasso A (2013). Antitumor activity of pimasertib, a selective MEK 1/2 inhibitor, in combination with PI3K/mTOR inhibitors or with multi-targeted kinase inhibitors in pimasertib-resistant human lung and colorectal cancer cells. Int J Cancer.

[161] Collisson EA, Trejo CL, Silva JM, Gu S, Korkola JE, Heiser LM (2012). A central role for RAF->MEK->ERK signaling in the genesis of pancreatic ductal adenocarcinoma. Cancer Discov.

[162] Viale A, Pettazzoni P, Lyssiotis CA, Ying H, Sanchez N, Marchesini M (2014). Oncogene ablation-resistant pancreatic cancer cells depend on mitochondrial function. Nature.

[163] Rusan M, Li K, Li Y, Christensen CL, Abraham BJ, Kwiatkowski N (2018). Suppression of Adaptive Responses to Targeted Cancer Therapy by Transcriptional Repression. Cancer Discov.

[164] Katoh M (2017). Canonical and non-canonical WNT signaling in cancer stem cells and their niches: Cellular heterogeneity, omics reprogramming, targeted therapy and tumor plasticity (Review). Int J Oncol.

[165] Rao G, Kim IK, Conforti F, Liu J, Zhang YW, Giaccone G (2018). Dasatinib sensitises KRAS-mutant cancer cells to mitogen-activated protein kinase kinase inhibitor via inhibition of TAZ activity. Eur J Cancer.

[166] Sharma V, Young L, Cavadas M, Owen K, Reproducibility Project (2016). Cancer B. Registered Report: COT drives resistance to RAF inhibition through MAP kinase pathway reactivation. Elife.

[167] Willems E, Dedobbeleer M, Digregorio M, Lombard A, Lumapat PN, Rogister B (2018). The functional diversity of Aurora kinases: a comprehensive review. Cell Div.

[168] Muzumdar MD, Chen PY, Dorans KJ, Chung KM, Bhutkar A, Hong E (2017). Survival of pancreatic cancer cells lacking KRAS function. Nat Commun.

[169] Yan L, Tu B, Yao J, Gong J, Carugo A, Bristow CA (2021). Targeting Glucose Metabolism Sensitizes Pancreatic Cancer to MEK Inhibition. Cancer Res.

[170] Tsubaki M, Takeda T, Noguchi M, Jinushi M, Seki S, Morii Y (2019). Overactivation of Akt Contributes to MEK Inhibitor Primary and Acquired Resistance in Colorectal Cancer Cells. Cancers (Basel).

[171] Halilovic E, She QB, Ye Q, Pagliarini R, Sellers WR, Solit DB (2010). PIK3CA mutation uncouples tumor growth and cyclin D1 regulation from MEK/ERK and mutant KRAS signaling. Cancer Res.

[172] Dai B, Meng J, Peyton M, Girard L, Bornmann WG, Ji L (2011). STAT3 mediates resistance to MEK inhibitor through microRNA miR-17. Cancer Res.

[173] Lee HJ, Zhuang G, Cao Y, Du P, Kim HJ, Settleman J (2014). Drug resistance via feedback activation of Stat3 in oncogene-addicted cancer cells. Cancer Cell.

[174] Anastas JN, Kulikauskas RM, Tamir T, Rizos H, Long GV, von Euw EM (2014). WNT5A enhances resistance of melanoma cells to targeted BRAF inhibitors. J Clin Invest.

[175] Tu B, Yao J, Ferri-Borgogno S, Zhao J, Chen S, Wang Q (2019). YAP1 oncogene is a context-specific driver for pancreatic ductal adenocarcinoma. JCI Insight.

[176] Moon JH, Hong SW, Kim JE, Shin JS, Kim JS, Jung SA (2019). Targeting beta-catenin overcomes MEK inhibition resistance in colon cancer with KRAS and PIK3CA mutations. Br J Cancer.

[177] Zanconato F, Cordenonsi M, Piccolo S (2016). YAP/TAZ at the Roots of Cancer. Cancer Cell.

[178] Lin L, Sabnis AJ, Chan E, Olivas V, Cade L, Pazarentzos E (2015). The Hippo effector YAP promotes resistance to RAF- and MEK-targeted cancer therapies. Nat Genet.

[179] Kapoor A, Yao W, Ying H, Hua S, Liewen A, Wang Q (2014). Yap1 activation enables bypass of oncogenic Kras addiction in pancreatic cancer. Cell.

[180] Shao DD, Xue W, Krall EB, Bhutkar A, Piccioni F, Wang X (2014). KRAS and YAP1 converge to regulate EMT and tumor survival. Cell.

[181] Kurppa KJ, Liu Y, To C, Zhang T, Fan M, Vajdi A (2020). Treatment-Induced Tumor Dormancy through YAP-Mediated Transcriptional Reprogramming of the Apoptotic Pathway. Cancer Cell.

[182] Suzuki R, Kikuchi S, Harada T, Mimura N, Minami J, Ohguchi H (2015). Combination of a Selective HSP90alpha/beta Inhibitor and a RAS-RAF-MEK-ERK Signaling Pathway Inhibitor Triggers Synergistic Cytotoxicity in Multiple Myeloma Cells. PloS one.

[183] Park KS, Oh B, Lee MH, Nam KY, Jin HR, Yang H (2016). The HSP90 inhibitor, NVP-AUY922, sensitizes KRAS-mutant non-small cell lung cancer with intrinsic resistance to MEK inhibitor, trametinib. Cancer Lett.

[184] Saei A, Palafox M, Benoukraf T, Kumari N, Jaynes PW, Iyengar PV (2018). Loss of USP28-mediated BRAF degradation drives resistance to RAF cancer therapies. J Exp Med.

[185] Hou P, Ma X, Zhang Q, Wu CJ, Liao W, Li J (2019). USP21 deubiquitinase promotes pancreas cancer cell stemness via Wnt pathway activation. Genes Dev.

[186] Hou P, Ma X, Yang Z, Zhang Q, Wu CJ, Li J (2021). USP21 deubiquitinase elevates macropinocytosis to enable oncogenic KRAS bypass in pancreatic cancer. Genes Dev.

[187] Sun Y, Daemen A, Hatzivassiliou G, Arnott D, Wilson C, Zhuang G (2014). Metabolic and transcriptional profiling reveals pyruvate dehydrogenase kinase 4 as a mediator of epithelial-mesenchymal transition and drug resistance in tumor cells. Cancer Metab.

[188] Bryant KL, Stalnecker CA, Zeitouni D, Klomp JE, Peng S, Tikunov AP (2019). Combination of ERK and autophagy inhibition as a treatment approach for pancreatic cancer. Nat Med.

[189] Kinsey CG, Camolotto SA, Boespflug AM, Guillen KP, Foth M, Truong A (2019). Protective autophagy elicited by RAF->MEK->ERK inhibition suggests a treatment strategy for RAS-driven cancers. Nat Med.

[190] Commisso C, Davidson SM, Soydaner-Azeloglu RG, Parker SJ, Kamphorst JJ, Hackett S (2013). Macropinocytosis of protein is an amino acid supply route in Ras-transformed cells. Nature.

[191] Yao W, Rose JL, Wang W, Seth S, Jiang H, Taguchi A (2019). Syndecan 1 is a critical mediator of macropinocytosis in pancreatic cancer. Nature.

[192] Ying H, Kimmelman AC, Lyssiotis CA, Hua S, Chu GC, Fletcher-Sananikone E (2012). Oncogenic Kras maintains pancreatic tumors through regulation of anabolic glucose metabolism. Cell.

[193] Kale J, Osterlund EJ, Andrews DW (2018). BCL-2 family proteins: changing partners in the dance towards death. Cell Death Differ.

[194] Hata AN, Yeo A, Faber AC, Lifshits E, Chen Z, Cheng KA (2014). Failure to induce apoptosis via BCL-2 family proteins underlies lack of efficacy of combined MEK and PI3K inhibitors for KRAS-mutant lung cancers. Cancer Res.

[195] Santamaria PG, Moreno-Bueno G, Cano A (2019). Contribution of Epithelial Plasticity to Therapy Resistance. J Clin Med.

[196] Peng DH, Kundu ST, Fradette JJ, Diao L, Tong P, Byers LA (2019). ZEB1 suppression sensitizes KRAS mutant cancers to MEK inhibition by an IL17RD-dependent mechanism. Sci Transl Med.

[197] Chen PY, Muzumdar MD, Dorans KJ, Robbins R, Bhutkar A, Del Rosario A (2018). Adaptive and Reversible Resistance to Kras Inhibition in Pancreatic Cancer Cells. Cancer Res.

[198] Obenauf AC, Zou Y, Ji AL, Vanharanta S, Shu W, Shi H (2015). Therapy-induced tumour secretomes promote resistance and tumour progression. Nature.

[199] Ruscetti M, Morris JPt, Mezzadra R, Russell J, Leibold J, Romesser PB (2020). Senescence-Induced Vascular Remodeling Creates Therapeutic Vulnerabilities in Pancreas Cancer. Cell.

[200] Brannon A 3rd, Drouillard D, Steele N, Schoettle S, Abel EV, Crawford HC (2020). Beta 1 integrin signaling mediates pancreatic ductal adenocarcinoma resistance to MEK inhibition. Sci Rep.

[201] Fedorenko IV, Wargo JA, Flaherty KT, Messina JL, Smalley KSM (2015). BRAF Inhibition Generates a Host-Tumor Niche that Mediates Therapeutic Escape. J Invest Dermatol.

[202] Brighton HE, Angus SP, Bo T, Roques J, Tagliatela AC, Darr DB (2018). New Mechanisms of Resistance to MEK Inhibitors in Melanoma Revealed by Intravital Imaging. Cancer Res.

[203] Boutin AT, Liao WT, Wang M, Hwang SS, Karpinets TV, Cheung H (2017). Oncogenic Kras drives invasion and maintains metastases in colorectal cancer. Genes Dev.

[204] Dow LE, O'Rourke KP, Simon J, Tschaharganeh DF, van Es JH, Clevers H (2015). Apc Restoration Promotes Cellular Differentiation and Reestablishes Crypt Homeostasis in Colorectal Cancer. Cell.

[205] Sui X, Chen Y, Liu B, Li L, Huang X, Wang M (2020). The relationship between KRAS gene mutation and intestinal flora in tumor tissues of colorectal cancer patients. Ann Transl Med.

[206] Briere DM, Li S, Calinisan A, Sudhakar N, Aranda R, Hargis L (2021). The KRASG12C Inhibitor MRTX849 Reconditions the Tumor Immune Microenvironment and Sensitizes Tumors to Checkpoint Inhibitor Therapy. Mol Cancer Ther.

[207] Poulikakos PI, Zhang C, Bollag G, Shokat KM, Rosen N (2010). RAF inhibitors transactivate RAF dimers and ERK signalling in cells with wild-type BRAF. Nature.

[208] Bhargava A, Anant M, Mack H, Reproducibility Project (2016). Cancer B, Reproducibility Project Cancer B. Registered report: Kinase-dead BRAF and oncogenic RAS cooperate to drive tumor progression through CRAF. Elife.

[209] Bhargava A, Pelech S, Woodard B, Kerwin J, Maherali N, Reproducibility Project (2016). Cancer B, et al. Registered report: RAF inhibitors prime wild-type RAF to activate the MAPK pathway and enhance growth. Elife.

[210] Paraiso KH, Fedorenko IV, Cantini LP, Munko AC, Hall M, Sondak VK (2010). Recovery of phospho-ERK activity allows melanoma cells to escape from BRAF inhibitor therapy. Br J Cancer.

[211] Hatzivassiliou G, Song K, Yen I, Brandhuber BJ, Anderson DJ, Alvarado R (2010). RAF inhibitors prime wild-type RAF to activate the MAPK pathway and enhance growth. Nature.

[212] Heidorn SJ, Milagre C, Whittaker S, Nourry A, Niculescu-Duvas I, Dhomen N (2010). Kinase-dead BRAF and oncogenic RAS cooperate to drive tumor progression through CRAF. Cell.

[213] Lito P, Pratilas CA, Joseph EW, Tadi M, Halilovic E, Zubrowski M (2012). Relief of profound feedback inhibition of mitogenic signaling by RAF inhibitors attenuates their activity in BRAFV600E melanomas. Cancer Cell.

[214] Hazar-Rethinam M, Kleyman M, Han GC, Liu D, Ahronian LG, Shahzade HA (2018). Convergent Therapeutic Strategies to Overcome the Heterogeneity of Acquired Resistance in BRAF(V600E) Colorectal Cancer. Cancer Discov.

[215] Corcoran RB, Andre T, Atreya CE, Schellens JHM, Yoshino T, Bendell JC (2018). Combined BRAF, EGFR, and MEK Inhibition in Patients with BRAF(V600E)-Mutant Colorectal Cancer. Cancer Discov.

[216] Migliardi G, Sassi F, Torti D, Galimi F, Zanella ER, Buscarino M (2012). Inhibition of MEK and PI3K/mTOR suppresses tumor growth but does not cause tumor regression in patient-derived xenografts of RAS-mutant colorectal carcinomas. Clin Cancer Res.

